# Impact of Dietary β-Mannans on the Intestinal-Nutritional Interface and Growth Performance of Chickens and Pigs: Challenges and Strategies via Exogenous β-Mannanase Based on Meta-Analysis

**DOI:** 10.3390/ani16193036

**Published:** 2026-09-26

**Authors:** Hyunjun Choi, Yesid Garavito-Duarte, Jeonghyeon Son, Sung Woo Kim

**Affiliations:** Department of Animal Science, North Carolina State University, Raleigh, NC 27695, USA; hchoi25@ncsu.edu (H.C.); yrgaravi@ncsu.edu (Y.G.-D.); json7@ncsu.edu (J.S.)

**Keywords:** β-mannans, β-mannanase, chickens, growth performance, intestinal health, pigs

## Abstract

The objectives of this review were to elucidate the physiological challenges induced by dietary β-mannans and to review the underlying modes of action of exogenous β-mannanase on the intestinal microbiota, intestinal health, nutrient utilization, and growth performance of chickens and pigs. The highly branched structure of β-mannans, particularly those with a high proportion of soluble β-mannans, can increase digesta viscosity and negatively modulate intestinal health by increasing the abundance of opportunistic pathogenic microbiota and reducing the abundance of bacteria associated with favorable metabolic outputs, such as short-chain fatty acids, in the small intestine. Furthermore, intact β-mannans are recognized by the intestinal immune system due to structural similarity to pathogen-associated molecular patterns in the small intestine, triggering unnecessary inflammatory responses. These combined factors damage intestinal structure and tight junction proteins and reduce nutrient utilization and growth performance in monogastric animals. Dietary β-mannanase supplementation can mitigate these challenges by breaking down the β-mannan structure. This enzymatic action reduces digesta viscosity and increases prebiotic manno-oligosaccharides in the small intestine, which positively modulates the diversity and abundance of intestinal microbiota, reduces immune responses, enhances intestinal barrier function and structure, and increases nutrient utilization. These gastrointestinal improvements enhance growth performance. Meta-analysis using a log response ratio scale (lnRR) showed that as dietary β-mannans increased, the mitigating effects of β-mannanase on intestinal health and growth performance of chickens became pronounced. When back-transformed to the percentage scale [(e^lnRR^ − 1) × 100], β-mannanase supplementation was estimated to reduce digesta viscosity in the small intestine by an average of 44.3% (slope on the log-scale = −0.584; 95% confidence interval: −65.0 to −11.3%; *p* < 0.05) and to increase average daily gain by an average of 5.23% (slope on the log-scale = 0.051; 95% confidence interval: 2.33 to 8.22%; *p* < 0.01) when dietary β-mannan content is 1% (DM basis). Further research, however, is warranted to refine model-derived estimates by considering variations in enzyme efficacy, non-standardized enzyme activity units, variable doses across studies, and the absence of a formal risk-of-bias assessment. In particular, the number of studies conducted in pigs was insufficient to allow meta-analysis, highlighting the need for further research to clarify β-mannanase efficacy across different dietary β-mannans. In conclusion, dietary β-mannanase serves as a valuable strategy that positively influences the intestinal microbiota, intestinal health, and nutrient utilization, thereby enhancing the growth performance of animals fed high β-mannan diets through the degradation of β-mannans.

## Simple Summary

Dietary β-mannans act as an anti-nutritional compound and represent a type of non-starch polysaccharide that impairs intestinal microbiota, intestinal health, nutrient utilization, and growth performance in monogastric animals. β-mannans, particularly those with a high mannose-to-galactose ratio and high solubility, increase digesta viscosity, thereby interfering with enzymatic digestion and hindering nutrient utilization in the small intestine. Furthermore, intact β-mannans possess structural similarities with microbial pathogen-associated molecular patterns, which may stimulate unnecessary innate immune responses in the small intestine, diverting available energy and nutrients from growth toward intestinal inflammation and cellular repair. Dietary β-mannanase supplementation mitigates these challenges by hydrolyzing the β-mannan backbone. This enzymatic cleavage breaks down the intact β-mannans, thereby reducing pathogen-associated molecular pattern-induced immune stimulation in the small intestine, reducing digesta viscosity, and generating prebiotic manno-oligosaccharides. These actions positively modulate the diversity and abundance of intestinal microbiota, enhance mucosal immunity and intestinal barrier function, and improve nutrient utilization and growth performance in monogastric animals. However, the negative impacts of β-mannans and the efficacy of the enzyme vary depending on the soluble β-mannan contents, which are largely determined by the mannose-to-galactose ratio. The negative impacts of dietary β-mannans on the intestinal microbiota, intestinal health, nutrient utilization, and growth performance of monogastric animals were elucidated, and the research investigating the underlying modes of action of exogenous β-mannanase was reviewed as a dietary strategy.

## 1. Introduction

Modern livestock production focuses on precision nutrition to maximize feed efficiency and minimize environmental impacts. Non-starch polysaccharide (NSP) in plant-based diets represents a primary factor limiting energy and nutrient utilization in monogastric animals [1,2]. Even in the corn-soybean meal (SBM)-based diets, NSP is present in high amounts, and its concentration can be substantially higher in alternative feedstuffs such as corn distillers dried grains with solubles, guar meal, palm kernel meal (PKM), and copra meal [3]. Although these co-products offer potential for cost reduction, their high NSP content often limits their inclusion levels in diet formulations due to their negative impacts on intestinal health and growth performance [4]. Among various NSP fractions, β-mannans, a linear polymer of mannose residues linked by β-1,4 glycosidic bonds, are recognized as an anti-nutritional compound (ANC) that impairs intestinal microbiota, intestinal health, and nutrient utilization across a wide range of feedstuffs [3]. In monogastric animal diets, β-mannans primarily exist as glucomannans found in cereal grain co-products or galactomannans in legume and oilseed meals. Their structural diversity, ranging from highly branched, soluble galactomannans with a low mannose-to-galactose ratio (M:G) in SBM to largely insoluble, linear structures with a high M:G in copra meal or PKM, influences digesta viscosity in the intestine and their susceptibility to enzymatic degradation.

The negative impacts of dietary β-mannans could occur in multiple ways. First, the branched structure of β-mannans increases digesta viscosity in the small intestine of monogastric animals [5,6]. The increased digesta viscosity reduces the passage rate of digesta, creating favorable conditions for the opportunistic pathogenic bacteria for their proliferation and altering the diversity and abundance of intestinal microbiota. Second, β-mannans possess a pathogen-associated molecular pattern (PAMP)-like structure, potentially triggering feed-induced innate immune responses in the small intestine [7,8]. Upon initial recognition through the activation of mannose receptors on resident macrophages, β-mannans can increase the expression of Toll-like receptor-4 in both intestinal epithelial cells and macrophages [9,10,11]. Regarding the immune-mediated impact of dietary β-mannans, the increased intestinal immune responses are accompanied by the increased production of pro-inflammatory cytokines and oxidative damage products, and the impaired intestinal structure, which diverts available energy and nutrients from growth toward epithelial tissue repair [12,13]. Separately, the high digesta viscosity in the small intestine reduces nutrient diffusion and limits the access of endogenous digestive enzymes to dietary substrates [13], collectively leading to a reduction in overall nutrient utilization. These two factors reduce the energy and nutrients available for growth in monogastric animals, including chickens and pigs, particularly when they are fed high β-mannan diets [5,8]. In this context, β-mannanase supplementation has been introduced to mitigate these challenges by hydrolyzing β-mannans. The hydrolysis of β-mannans in the small intestine reduces digesta viscosity, generates manno-oligosaccharides (MOS), and attenuates their PAMP-like activity [8,14], thereby mitigating the negative impacts on intestinal microbiota, intestinal health, nutrient utilization, and growth in chickens [6,8] and pigs [5,12].

Previous research has predominantly focused on nutrient utilization, and despite a recent growing interest in intestinal microbiota and intestinal health, comprehensive information regarding how β-mannanase bridges the gap between intestinal health and growth performance remains limited [15,16] (Figure 1). Therefore, the objectives of this review were to investigate the negative impacts of dietary NSP, including β-mannans, on the intestinal microbiota, intestinal health, nutrient utilization, and growth performance, and to elucidate the modes of action of dietary β-mannanase supplementation as a targeted dietary strategy for the intestinal microbiota, intestinal health, nutrient utilization, and growth performance in both chickens and pigs.

## 2. Materials and Methods

### 2.1. Literature Search, Eligibility Criteria, and Study Selection

A literature search was conducted using PubMed and Google Scholar with keywords including β-mannans, β-mannanase, intestinal health, digesta viscosity, nutrient digestibility, growth performance, chickens, and pigs. The initial search yielded approximately 316 papers for chickens and 338 papers for pigs. Of these, 46 papers for chickens and 41 papers for pigs were screened based on title and abstract. After full-text review according to the population, intervention, comparator, outcome, and study design (PICOS) framework [17], 41 papers for chickens and 26 papers for pigs were finally selected. The summary of the inclusion and exclusion criteria on the PICOS framework is presented as follows. The targeted population was restricted to commercial breeds of pigs (*Sus domesticus*) and chickens (*Gallus gallus domesticus*) commonly utilized in the livestock industry. For dietary intervention, eligible studies evaluated the effects of β-mannanase supplementation across different levels, sources, or enzymatic activities in diets. Studies combining β-mannanase with other feed additives or therapeutic treatments, such as antibiotics, were excluded if they confounded its specific efficacy; however, studies were included if their treatment structure allowed for the independent evaluation of the main effect of β-mannanase (factorial treatment arrangement). In addition, the studies involving pathogenic bacterial challenges were excluded from the meta-analysis applied in this review as infection status could introduce confounding effects on the measured parameters. Included studies were required to have a control group fed a basal diet without β-mannanase supplementation, although additional comparators testing different doses or forms were also acceptable. Studies lacking a non-supplemented negative control group were excluded. Furthermore, studies had to report at least one in vivo parameter related to intestinal health and growth, including digesta viscosity in the small intestine, diversity and abundance of intestinal microbiota, immune responses, oxidative damage products, intestinal barrier function and structure, nutrient utilization, or growth performance. Studies limited strictly to in vitro or ex vivo evaluations were excluded. Regarding experimental design, only controlled experimental studies employing a randomized complete block design or Latin square design, such as blocking by initial body weight (IBW), age, sex, or period were selected, whereas completely randomized designs, observational studies, and studies lacking clear methodologies were excluded. Finally, only studies published in English were considered eligible for inclusion.

### 2.2. Data Collection and Processing Based on Summary of Selected Studies

Using predefined eligibility criteria, data collection was performed independently by 2 reviewers using a standardized data collection form. For each study, the following information was recorded: author, year of publication, animal species, IBW or age at the start of the study, dietary β-mannan contents, enzymatic units and levels of β-mannanase supplemented, experimental period, and measured parameters including digesta viscosity in the small intestine, diversity and abundance of intestinal microbiota, intestinal health, nutrient utilization, or growth performance. Discrepancies between reviewers were resolved through consensus, and the final collected dataset was thoroughly verified to ensure accuracy and completeness. Data were systematically structured to facilitate comparison and synthesis across studies. It is well recognized that the contents of soluble NSP and their relative proportion within the total non-starch polysaccharide (tNSP) fraction are the primary determinants of digesta viscosity in the small intestine of animals [18,19,20]. However, due to the limited availability of specific data regarding soluble β-mannans across the evaluated feedstuffs, a comprehensive isolation of the soluble fraction was not feasible in this review. Furthermore, in plant-based feedstuffs, β-mannans naturally co-exist with a variety of other NSP compounds, which may cause potential confounding effects when isolating the specific physiological impacts of β-mannans alone [21,22,23]. Thus, to establish a consistent basis for comparing results among different studies, both dietary β-mannans and tNSP contents were presented in tables. Additionally, measured composition of dietary β-mannans and tNSP were used when reported in the previous study. Otherwise, dietary contents of both β-mannans and tNSP were determined by multiplying the inclusion rate of each feedstuff by its respective value obtained from Supplementary Table S1 and summing these values. Accordingly, studies utilizing feedstuffs not listed in Supplementary Table S1 were excluded from the analysis. For studies involving phase-feeding, the dietary β-mannans and tNSP contents were averaged for general presentation. For quantitative evaluation of the impacts of β-mannans on animals, response variables such as digesta viscosity in the small intestine and growth performance were determined as percentage changes relative to the control group for comparison across studies. The percentage change of the response variable was determined in the treatment group relative to the control group based on the following equation:

ΔResponse variable (%) = (mean value of response variable in the treatment group − mean value of response variable in the control group)/mean value of response variable in the control group × 100

Statistical difference is denoted by asterisks, where * and ** represent a statistical tendency (0.05 ≤ *p* < 0.10) and significance (*p* < 0.05), respectively (Tables 1 and 3). For other parameters where quantitative evaluation was limited due to heterogeneity in measurement units or reporting methods, the responses to β-mannans or β-mannanase were qualitatively summarized based on statistical significance (increase or decrease), tendency, or no response (Tables 2 and 4–6).

### 2.3. Statistical Analysis Based on Regression

For meta-analysis, datasets were analyzed using the Proc Mixed procedure of SAS (SAS Inst., Cary, NC, USA) to evaluate log response ratio (lnRR) of digesta viscosity in the small intestine (lnRR_Digesta viscosity_) and average daily gain (lnRR_ADG_) of animals fed diets with varying contents of dietary β-mannans and β-mannanase supplementation, relative to the group without β-mannanase supplementation. For digesta viscosity in the small intestine of chickens and pigs, the search initially yielded 17 papers for chickens and 8 papers for pigs, from which 8 papers (8 studies) and 5 papers (6 studies) were selected based on the PICOS framework after reading each article, respectively. For ADG, the search yielded 49 papers for chickens and 39 papers for pigs, from which 28 papers (30 studies) and 19 papers (27 studies) were selected, respectively (Tables S3 and S4). The selected peer-reviewed articles published between 2000 and 2026 were retained for meta-analysis. The meta-analysis was initially intended to determine the effects of β-mannanase supplementation on lnRR of digesta viscosity in the small intestine and ADG in both chickens and pigs. However, the number of eligible studies for pigs was limited: for digesta viscosity, only 5 papers (6 studies, 11 observations) met the inclusion criteria, and for growth performance, 19 papers consisting of 14 studies (21 observations) in the nursery phase and 13 studies (27 observations) in the growing-finishing phase were available (Table S4). Given this limited number of studies relative to the high inherent variation across growth stages and experimental conditions, the statistical model failed to converge or to yield significant estimates for pigs. Therefore, the meta-analysis was focused on chickens. To enhance statistical rigor and normalize skewed effect sizes across studies for the meta-analysis [24], the digesta viscosity in the small intestine and average daily gain (ADG) of chickens in the meta-analysis were determined as lnRR instead of percentage changes, calculated as:

lnRR_Digesta viscosity in chicken_ = ln (mean value of digesta viscosity in the β-mannanase group/mean value of digesta viscosity in the control group without β-mannanase supplementation)

lnRR_ADG in chicken_ = ln (mean value of ADG in the β-mannanase group/mean value of ADG in the control group without β-mannanase supplementation)

Expressing effect sizes as lnRR provides an additive linear scale with a known sampling distribution, thereby increasing accuracy across studies with varying baseline values [25]. To account for differences in statistical precision among observations, inverse-variance weighting was included in the model [26]. The weighting factor was calculated as:

Weighting factor = *n*/CV^2^

where *n* represents the sample size (number of replicates in the study) and CV is the coefficient of variation in the observation. This weighting framework assigns greater weight to studies with larger sample sizes and lower relative variability [26]. Potential multicollinearity between dietary predictors, such as dietary β-mannans and tNSP, was evaluated using the Proc CORR and REG procedures to determine Pearson correlation coefficient (r) and variance inflation factor (VIF) [27]. To account for study variability, study was included in the model as a random effect [28,29,30]. Between-study heterogeneity was defined as the proportion of total variance attributed to the random effect of study, calculated as σ^2^ study/(σ^2^ study + σ^2^ residual), analogous to the intraclass correlation coefficient (ICC) used in conventional meta-analytic frameworks [31]. A heterogeneity ratio ≥ 50% was considered indicative of substantial between-study heterogeneity [31]. When heterogeneity was substantial, a sensitivity analysis using influence diagnostics (Cook’s distance and restricted likelihood distance) was additionally performed to identify studies exerting disproportionate leverage on the pooled estimate, and the corresponding fixed-effect estimates were examined for consistency upon their exclusion [32]. Publication bias and small-study effects were visually evaluated using a funnel plot of lnRR against its standard error and further examined using Egger’s regression asymmetry test [33].

Because lnRR values are expressed on a logarithmic scale, they are not directly interpretable in biological terms. Thus, the lnRR values obtained from the meta-analysis were back-transformed into percentage change to allow for a more direct, biologically understandable interpretation of the treatment effect, using the following equation:

∆Digesta viscosity or ADG (%) = (e^lnRR^ − 1) × 100

This back-transformation converts the lnRR into the corresponding percentage change between the β-mannanase group and the control group without β-mannanase supplementation, thereby allowing the meta-analysis results to be expressed and interpreted in the same manner as conventional percentage change values.

## 3. Structure and Characterization of Non-Starch Polysaccharide and β-Mannans in Diets

### 3.1. Chemical and Structural Diversity of NSP and β-Mannans in Diets

Plant-based feedstuffs are widely used in cost-effective diet formulations for monogastric animals; however, they contain various NSP fractions linked by β-glycosidic bonds [23]. Because chickens and pigs lack endogenous enzymes to hydrolyze these bonds [34], NSP acts as ANC. These NSP fractions increase digesta viscosity and alters the available nutrient composition in the small intestine, shifting the substrate composition and modulating the diversity and abundance of intestinal microbiota [18,20,35]. Therefore, an understanding of the structural characterization of NSP fractions is critical, as they are highly relevant to understanding their influence on the intestinal microbiota, intestinal health, nutrient utilization, and growth in monogastric animals.

Non-starch polysaccharide in feedstuffs is mainly located within plant cell walls, comprising the middle lamella alongside the primary and secondary cell walls [36], and is broadly classified into soluble and insoluble NSP fractions [37]. Their chemical and structural diversity arises from the variety of monosaccharide units and the complexity of their glycosidic linkages. The building blocks of NSP include pentoses (arabinose and xylose), hexoses (glucose, galactose, and mannose), 6-deoxyhexoses (rhamnose), and uronic acids (glucuronic and galacturonic acids) [3,38]. These monosaccharides exist in both pyranose and furanose ring forms and are linked through α or β orientations at various hydroxyl groups, resulting in an immense number of three-dimensional shapes and functional surfaces [38]. These structural variations in NSP, such as monosaccharide composition, linkage types, and degree of branching, determine their solubility. Collectively, soluble NSP fractions absorb water to increase digesta viscosity in the small intestine, which reduce the interaction between digestive enzymes and nutrients, whereas insoluble NSP fractions physically restrict the exposure of digestible nutrients within the plant cell wall matrix [22].

As a major heterogeneous fraction of NSP, hemicellulose contains β-mannans, which are particularly notable due to their unique distribution in protein-rich feedstuffs and their branched structure [3]. These heteropolysaccharides (e.g., galactomannans) are distinguished from other hemicelluloses, such as xylans, which possess different branching patterns and sugar compositions [7,39]. β-mannans are present at high concentrations in feedstuffs such as SBM, guar meal, and corn distillers dried grains with solubles, existing primarily as glucomannans and galactomannans.

Glucomannans feature a heteropolymeric backbone of alternating D-glucose and D-mannose units linked by β-1,4 bonds, which are commonly found in cereal grain co-products [3]. In contrast, galactomannans consist of a homopolymeric β-1,4-mannose backbone heavily branched with α-1,6-linked D-galactose side chains [3]. As galactomannans are predominant in legume-derived feedstuffs like SBM, their solubility and potential to increase digesta viscosity in the small intestine of animals are mainly determined by the M:G. Specifically, SBM (M:G = 1.8:1) and guar meal (M:G = 2:1) have highly branched structures. This extensive branching creates steric hindrance between the β-1,4-D-mannan backbones, which prevents the formation of inter-chain hydrogen bonds and maintains high solubility [40]. In contrast, β-mannans in copra meal and PKM feature a linear backbone with fewer side chains, having a 13:1 to 20:1 M:G, which leads to lower water solubility. In short, a low M:G corresponds to high branching, high solubility, and a relatively strong impact on increasing digesta viscosity in the small intestine of animals, whereas a high M:G reflects low branching, low solubility, and a relatively small impact. Therefore, the efficiency of their enzymatic degradation can vary depending on the feedstuffs.

### 3.2. Physicochemical Properties of Soluble NSP Fractions and β-Mannans: The Impact on Digesta Viscosity Related to Intestinal Health of Animals

The solubility of NSP determines their functional properties within the gastrointestinal tract [41,42]. The insoluble NSP fraction in diets shows a high degree of lignification and is the less fermentable fraction in the gastrointestinal tract of monogastric animals, increasing the bulk of the digesta and leading to a faster passage rate [43]. In contrast, the soluble NSP fraction in diets is less lignified and increases digesta viscosity in the small intestine, thereby reducing nutrient utilization in chickens and pigs [35,44]. Because the soluble NSP content is lower than that of the insoluble NSP fraction [3], its impact on nutrient utilization has often been overlooked. However, digesta viscosity independently influences the decrease in the ileal digestibility of gross energy (GE) and nutrients, with studies even showing that the effects of digesta viscosity are greater than those of the tNSP content regarding nutrient utilization in chickens [45] and pigs [20].

The negative impact of increased digesta viscosity, induced by soluble NSP fractions, is more pronounced in the small intestine than in the large intestine of the animals [19,20]. Interestingly, a previous study investigating the relationship between NSP fractions and the total tract digestibility of GE and nutrients in animals [37] showed that insoluble NSP fractions had a stronger negative correlation than soluble NSP fractions in diets. This deviation can be attributed to the location-specific roles of NSP and the physiological maturity of the animals. The proportion of insoluble NSP fractions in the feces is greater than that in the ileal digesta, and the total tract digestibility of soluble NSP fractions is greater than that of insoluble NSP fractions in animals [46,47,48]. This indicates that soluble NSP fractions are more easily fermented and degraded in the hindgut than insoluble NSP fractions, whereas their presence within the small intestine before reaching the hindgut is particularly negative, as the increased digesta viscosity hinders nutrient utilization. Given that the majority of nutrient digestion and absorption occurs in the small intestine, there is growing interest in how increased digesta viscosity, which is substantially influenced by soluble NSP fractions, affects the intestinal microbiota, intestinal health, and nutrient utilization of monogastric animals.

Among soluble NSP fractions, β-mannans with highly branched structures can have a pronounced impact on digesta viscosity in the small intestine, which is clearly reflected in in vivo responses (Table 1). Soybean meal contains highly branched galactomannans characterized by a low M:G (approximately 1.8:1). Due to this branched structure, which has a high water-holding capacity [40], an increase in β-mannans from 0.35 to 0.53% in the diet [dry matter (DM) basis] resulting from higher SBM inclusion was sufficient to increase digesta viscosity by up to +20.4% in the jejunum of pigs [5] (Table 1). Additionally, in pigs, increasing β-mannans from 0.40 to 1.19% in the diet using guar gum (a galactomannan-rich source), increased digesta viscosity by +49.7% in the jejunum [49], and a further increase from 0.42 to 6.02% β-mannans in the diet increased digesta viscosity by +266.5% in the ileum [50] (DM basis). Similarly, when chickens were fed galactomannan-rich sources such as guar gum or guar germ, these compounds induced a dose-dependent increase in digesta viscosity in the small intestine, as β-mannans increased from 0.49 to 0.98% in the diet with guar gum [51] and from 0.59 to 1.06% in the diet with guar germ [52] (DM basis). This resulted in increased digesta viscosity up to +658.9% in the jejunum [51] and up to +1480.6% in the ileum [52].

Copra meal and PKM, however, possess high total β-mannan contents but feature structures with fewer side chains and high M:G (13:1 to 20:1). The high M:G in copra meal and PKM leads to a lower proportion of soluble β-mannans to total β-mannans, thus despite β-mannans accounting for a high proportion of tNSP in copra meal (around 42.2%), the soluble β-mannans proportions of total β-mannans remain at around 5% [3,53]. Due to the structural difference in copra meal and PKM compared to feedstuffs having highly branched β-mannans, these feedstuffs may show a relatively lower impact on digesta viscosity in the small intestine of animals. A previous study reported that increasing β-mannans from 0.49 to 15.24% in the diet (DM basis) via a higher inclusion rate of copra meal and PKM linearly decreased jejunal digesta viscosity by up to 31.2% [21]. Although the high increase in dietary tNSP from 10.8 to 23.4% in that same study (DM basis) may have induced a confounding effect on digesta viscosity, the absence of an increase in digesta viscosity in the small intestine indicates that linear β-mannans with high M:G show high insolubility and have a relatively low water-holding capacity to increase digesta viscosity [21]. Collectively, despite the high total β-mannan contents in copra meal and PKM, they may contribute relatively less to digesta viscosity in the small intestine of monogastric animals, compared to the highly branched, highly soluble galactomannans found in SBM or guar meal.

**Table 1:** Digesta viscosity in the small intestine of chickens and pigs fed diets high in β-mannans ^1,2^.

Age or IBW	NSP Composition ^3^ (% in DM Diet)		Experimental Period (d)	Digesta Viscosity (% Change)	Reference
	β-Mannans	Total NSP (tNSP)			
Chicken (d)					
8	0.65	13.4	34	-	[54]
	0.92	13.3		+16.9% (ileum)	
	1.19	13.2		+40.0% **	
	1.46	13.1		+69.4% **	
1	0.45	10.7	14, 28, 42	-	[55]
	0.60	10.8		+18.4% * at d 14; +58.1% ** at d 28; +11.0% at d 42 (ileum)	
	0.75	11.0		+61.6% ** at d 14, +164.8% ** at d 28; +27.1% at d 42 (ileum)	
4	0.49	10.8	10	-	[21]
	3.44	13.4		−5.6% ** (jejunum)	
	9.36	18.6		−21.4% **	
	15.24	23.4		−31.2% **	
7	0.49	11.2	14	-	[51]
	0.58	12.5		−7.9% (jejunum)	
	0.98	12.9		+658.9% **	
1	0.59	12.7	21	-	[52]
	0.82	12.2		+126.1% ** (ileum)	
	0.94	12.0		+448.9% **	
	0.59	12.7		-	
	0.82	12.7		+377.8% ** (ileum)	
	1.06	12.8		+1480.6% **	
Pig (kg)					
6.2	0.35	8.4	37	-	[5]
	0.53	11.3		+20.4 * (jejunum)	
9.5	0.40	11.3	12	-	[49]
	1.19	11.8		+49.7% ** (jejunum)	
27.0	0.42	14.8 ^4^	17	-	[50]
	6.02	23.1 ^4^		+266.5% ** (ileum)	

IBW = initial body weight; DM = dry matter; NSP = non-starch polysaccharide. ^1^ Asterisk marks (*,**) represent statistical tendency (*p* < 0.10) and significant difference (*p* < 0.05), respectively. ^2^ The percentage increase or decrease in digesta viscosity in the small intestine was determined as: [(mean value in the high β-mannans group − mean value in the control group)/mean value in the control group] × 100, and all percentage changes were recalculated in this study based on the reported values from the original studies. ^3^ The dietary contents of both β-mannans and tNSP were determined by multiplying the inclusion rate of each feedstuff by its respective value obtained from Supplementary Table S1 and summing these values. ^4^ The chemical composition was an analyzed value.

### 3.3. Physiological Modulation: Intestinal Microbiota, Intestinal Health, Nutrient Utilization, and Growth Performance

In monogastric nutrition, these branched β-mannans act as ANC [5,52]. Beyond their physical effect of increasing digesta viscosity, β-mannans independently trigger innate immune responses in the small intestine [7,9,10]. The increased immune responses lead to unnecessary energy and nutrient expenditure for epithelial cell repair in the small intestine, ultimately reducing the growth performance of chickens [8,52] and pigs [5]. Therefore, elucidating how β-mannans and co-existing NSP in feedstuffs reduce nutrient utilization by increasing digesta viscosity, as well as the direct impact of β-mannans on intestinal inflammation and structure, is essential to mitigate negative impacts of β-mannans on the intestinal microbiota, intestinal health, nutrient utilization, and the growth performance of monogastric animals (Table 2).

**Table 2:** Metabolites and intestinal microbiota, immune responses, intestinal barrier function and structure, and nutrient utilization in chickens and pigs fed diets high in β-mannans. Changes were indicated using ↑ (increase) and ↓ (decrease).

Age or IBW	Difference ^1^ (% in DM Diet)		Experimental Period (d)	Result	Reference
	β-Mannans	Total NSP (tNSP)			
Metabolite and intestinal microbiota					
Chicken (d)					
14	0.55 vs. 2.14	12.4 vs. 13.8	14	Content: ↓ acetate, ↓ total SCFA, ↑ lactic acid; Index: ↓ Shannon, ↓ Simpson; RA: ↑ *Lactobacillus*, ↓ *Clostridium*, ↓ Anaerostipes (cecum)	[56]
Pig (kg)					
6.2	0.35 vs. 0.53	8.4 vs. 11.3	37	RA: ↓ Lactobacillaceae, ↑ Helicobacteraceae (jejunum)	[5]
9.5	0.40 vs. 1.19	11.3 vs. 11.8	12	Content: ↓ lactic acid; Count: ↑ *Escherichia coli*, ↑ *Streptococci* (jejunum)	[49]
	0.36 vs. 0.81	11.3 vs. 11.6		Content: ↓ acetate, ↑ butyrate; Count: ↑ *Escherichia coli*, ↑ *Streptococci* (jejunum)	
27.0	0.42 vs. 6.02	9.9 vs. 15.5	17	Content: ↑ total anaerobes, ↑ total aerobes, ↑ *Lactobacilli*, ↑ *Enterobacteria* (ileum)	[50]
Immune response					
Chicken (d)					
14	0.10 vs. 1.70	3.2 vs. 4.7	14	mRNA expression: ↑ CCLi2, ↑ IL-6, ↑ IL-8, ↓ IgA (ileum); Content: ↑ α-1GP, ↑ SAA (serum)	[56]
	0.10 vs. 3.30, 4.90	3.2 vs. 6.3, 7.8		mRNA expression: ↑ CCLi2, ↑ IL-6, ↑ IL-1, ↑ IL-8 (ileum)	
Pig (kg)					
6.2	0.35 vs. 0.52	9.2 vs. 11.7	42	Content: ↑ IL-8 (jejunum)	[57]
Intestinal barrier function and structure					
Chicken (d)					
1	0.59 vs. 1.04	7.1 vs. 7.0	35	↓ VH, ↓ VH:CD (ileum)	[58]
14	0.55 vs. 2.14	12.4 vs. 13.8	14	↑ FITC-dextran (serum)	[56]
Pig (kg)					
6.2	0.35 vs. 0.53	8.4 vs. 11.3	37	mRNA expression: ↓ ZO-1 (jejunum)	[5]
9.5	0.40 vs. 1.19	11.3 vs. 11.8	12	↓ VH:CD (jejunum)	[49]
Nutrient utilization					
Chicken (d)					
4	0.49 vs. 3.44	10.8 vs. 13.4	10	↓ ATTD of DM and CP	[21]
	0.49 vs. 9.36, 15.24	10.8 vs. 18.6, 23.4		↓ ATTD of DM and CP, AME	
7	0.49 vs. 0.98	12.5 vs. 12.8	14	↓ ATTD of GE	[51]
1	0.55 vs. 0.95 to 2.15	11.9 vs. 12.3 to 13.5	14	↓ AME	[59]
11	0.55 vs. 2.14	12.4 vs. 13.8	10	↓ AID and SID of all essential AA and non-essential AA	[56]
14	0.10 to 4.90	3.2 to 7.8	14	(linear) ↓ ATTD of DM	
Pig (kg)					
6.2	0.35 vs. 0.52	9.2 vs. 11.7	42	↓ AID of EE	[57]
27.0	0.42 vs. 6.02	9.9 vs. 15.5	17	↓ AID and ATTD of GE, ↓ ATTD of CP, ↓ DE	[50]
34.7	2.02 to 7.92	16.1 to 19.7	10	(linear) ↓ ATTD of CP	[60]
34.9	0.61 ^2^ vs. 2.52 ^2^	12.2 vs. 13.8	42	↓ ATTD of GE, ↓ ATTD of β-mannans	[61]

AA = amino acid; AID = apparent ileal digestibility; α-1GP = alpha-1-acid glycoprotein; AME = apparent metabolizable energy; ATTD = apparent total tract digestibility; CP = crude protein; DM = dry matter; EE = ether extract; VH = villus height; VH:CD = villus height-to-crypt depth ratio; FITC = fluorescein isothiocyanate; GE = gross energy; IL-1 = interleukin-1; IL-8 = interleukin-8; CCLi2 = K203 macrophage chemokine; RA = relative abundance; SAA = serum amyloid A; SCFA = short-chain fatty acids; SID = standardized ileal digestibility; ZO-1 = zonula occludens-1. ^1^ The dietary contents of both β-mannans and tNSP were determined by multiplying the inclusion rate of each feedstuff by its respective value obtained from Supplementary Table S1 and summing these values. ^2^ The chemical composition was an analyzed value.

Dietary soluble NSP fractions, including arabinoxylans, β-glucans, and β-mannans could have a negative impact on intestinal health of chickens and pigs, mainly because increased jejunal digesta viscosity negatively alters the diversity and abundance of intestinal microbiota [35,62]. Specifically, this viscous matrix promotes the overgrowth of opportunistic pathogenic bacteria in the small intestine [18]. The negative impact of soluble NSP fractions becomes pronounced during environmental or infectious challenges, such as *Eimeria* and *Clostridium perfringens* challenges in chickens [63,64] or an F18^+^ *Escherichia coli* challenge in nursery pigs [65]. In contrast, the inclusion of a moderate amount of insoluble NSP fractions can mitigate the negative impacts of infectious challenges on intestinal health and growth performance by stimulating intestinal peristalsis and shortening the retention time of pathogenic bacteria in the intestine [66,67,68]. Previous studies have reported that the increased digesta viscosity induced by soluble NSP fractions, including β-mannans, promotes the overgrowth of opportunistic pathogenic bacteria such as *Escherichia coli*, *Streptococci*, and Helicobacteraceae in the jejunum of pigs [5,49], often at the expense of populations like Lactobacillaceae [5]. These microbial shifts are accompanied by a reduction in microbial diversity indices and a decrease in the concentrations of metabolites such as acetate and other short-chain fatty acids (SCFA) in the cecum of chickens [56], as well as reduced lactic acid content in the jejunum of pigs [49]. In chickens, the high DM content in ileal digesta could amplify the negative impact of soluble NSP fractions, including soluble β-mannans, on digesta viscosity, intestinal health, and nutrient utilization. Although both soluble and insoluble NSP fractions decrease nutrient utilization in monogastric animals, increased digesta viscosity associated with soluble NSP fractions has been identified as an important factor contributing to reduced growth performance of animals. The negative impacts of soluble NSP fractions can be further influenced by the age of animals. In young monogastric animals, immature digestive and immune systems make them more vulnerable to the digesta viscosity and anti-nutritional effects of soluble NSP fractions compared with older animals [69,70].

Beyond the negative impact of soluble NSP fractions on digesta viscosity, β-mannans stand out from other NSP fractions like arabinoxylans and cellulose because of the unique branched structure of β-mannans that may stimulate intestinal immune responses of monogastric animals [7,39]. Specifically, β-mannans exert various negative impacts on the intestinal health of chickens and pigs, concurrently disrupting the diversity and abundance of intestinal microbiota, inducing unnecessary intestinal immune responses, impairing barrier function and structure, and reducing nutrient utilization (Table 2). This occurs likely because intact β-mannans are structurally similar to the PAMPs found on the cell walls of certain bacteria [71,72,73]. Thus, β-mannans can be recognized by pattern recognition receptor (PRR) present on macrophages, primarily C-type lectin receptors such as the mannose receptor [73,74]. The capacity of β-mannans to activate the intestinal immune responses stems from their complex branched structure [75]. This branching allows multiple mannose residues to bind simultaneously to several carbohydrate recognition domains on the mannose receptor, thereby enhancing the overall binding affinity [10,76]. Such binding stimulates the production of inflammatory markers in the intestine of animals [77]. This includes the upregulation of pro-inflammatory cytokines, such as IL-1, IL-6, and IL-8, and the CCLi2 (K203 macrophage chemokine) in the ileal mucosa of chickens [56]. This localized inflammation often extends systemically, as indicated by increased serum concentrations of acute-phase proteins like α1-acid glycoprotein and serum amyloid A (SAA). Furthermore, dietary β-mannans negatively affect intestinal barrier function and structure [58]. Increased levels of β-mannans from 0.36 to 1.16% in the diet (DM basis) lead to a reduced villus height (VH) and villus height-to-crypt depth ratio (VH:CD) [49], along with the downregulation of tight junction proteins like zonula occludens-1 (ZO-1) when β-mannans increase from 0.35 to 0.53% in the diet [5] (DM basis). The structural damage to the intestinal epithelium can be further evidenced by elevated serum concentrations of fluorescein isothiocyanate (FITC)-dextran [56]. The increased digesta viscosity also limits the diffusion of digestive enzymes and nutrients, while the chronic reaction of innate immune pathways, triggered by the PAMP-like structure of β-mannans, results in a futile physiological response [78].

In addition to the anti-nutritional effects of dietary β-mannans on digesta viscosity, intestinal microbiota, and intestinal health, dietary β-mannans reduce GE and nutrient utilization in both chickens and pigs. Multiple studies have consistently reported that increased dietary β-mannans reduced the apparent ileal digestibility (AID) and apparent total tract digestibility (ATTD) of GE and nutrients [51,57,61] (Table 2). Importantly, the negative impact on nutrient utilization occurs independently of tNSP levels. For instance, even in diets where tNSP contents remain relatively low or comparable (11.9 to 12.8% in the diet; DM basis), an increase in dietary β-mannans from 0.50 to 0.93% reduced apparent metabolizable energy (AME) and ATTD of GE in chickens [51] (DM basis). Similarly, in pigs, increasing β-mannans from 0.35 to 0.52% in the diet reduced the AID of nutrients under relatively low tNSP change from 9.3 to 11.6% in the diet [57] (DM basis). Furthermore, purified β-mannans (up to 5.97% of dietary β-mannans supplemented via guar gum; DM basis) reduced the AID and ATTD of GE and CP of pigs [61]. These findings indicate that β-mannans show a potent, independent negative impact on nutrient utilization across species, regardless of whether tNSP content increases. Collectively, the reduced nutrient utilization and the diversion of energy and nutrients induced by β-mannans toward unnecessary intestinal immune responses and epithelial tissue repair [79] lead to reduced growth performance of chickens [8,52] and pigs [5,57] (Table 3).

**Table 3:** Growth performance in chickens and pigs fed diets high in β-mannans ^1,2^.

Age or IBW	NSP Composition ^3^ (% in DM Diet)		Experimental Period (d)	ME (kcal/kg)	Growth Performance (% Change)			Reference
	β-Mannans	Total NSP (tNSP)			ADG	ADFI	G:F	
Chicken (d)								
1	0.59	12.7	21	3075	-	-	-	[52]
	0.82	12.2			−1.21	1.58	−2.74	
	0.94	12.0			−4.98	5.73	−10.13	
	0.59	12.7			−11.79 **	−6.2	−5.96	
	0.82	12.7			−22.56 **	−8.92	−14.97 **	
1	0.59	7.1	35	3100	-	-	-	[58]
	1.04	7.0			−4.33 **	3.81	−7.85 **	
8	0.65	13.4	34	3000	-	-	-	[54]
	0.92	13.3			−0.59	−1.4	0.82	
	1.19	13.2			−1.62	1.56	−3.13	
	1.46	13.1			−8.98 **	−3.04	−6.13 **	
1	0.55	11.9	14	3150	-	-	-	[59]
	0.95	12.3			−7.24 **	−6.38	−0.93	
	1.35	12.7			−4.71 **	−1.96	−2.81 **	
	2.15	13.5			−14.97 **	3.8	−18.08 **	
1	0.45	10.7	42	3234	-	-	-	[55]
	0.60	10.8			−1.53	−0.20	−1.34 **	
	0.75	11.0			−1.37	2.19	−3.48 **	
7	0.49	11.2	14	3000	-	-	-	[51]
	0.58	12.5			4.26 **	3.66	0.57 **	
	0.98	12.9			−10.02 **	1.25	−11.13 **	
4	0.49	10.8	10	3200	-	-	-	[21]
	3.44	13.4			−30.93 **	−13.92 **	−19.76 **	
	9.36	18.6			−29.77 **	−13.19 **	−19.10 **	
	15.24	23.4			−50.50 **	−26.14 **	−32.99 **	
Pig (kg)								
6.2	0.35	8.4	37	3400	-	-	-	[5]
	0.53	11.3			−11.68 *	−13.99 **	2.68	
6.2	0.35	9.2	42	3330	-	-	-	[57]
	0.52	11.7			−1.37	−5.27	4.12 *	
6.6	0.64	13.0	28	3390	-	-	-	[80]
	3.59	15.6			−2.63 **	−3.70 **	1.11	
6.9	0.36	8.1	42	3510	-	-	-	[81]
	6.45	15.2			5.52 *	10.91 **	−4.85	
26.0	0.42	9.9	14	N/A	-	-	-	[50]
	6.02	15.5			−16.97 **	−15.27 **	−2.01	
31.2	2.02	16.1	84	3265	-	-	-	[60]
	3.49	17.0			0.24	−1.52	1.78	
	4.97	17.9			−2.38	−5.50	3.30	
	6.44	18.8			0.36	−1.27	1.65	
	7.92	19.7			−2.73	−2.79	0.06	
34.9	0.61 ^4^	12.2	42	3303	-	-	-	[61]
	2.52 ^4^	13.8			−2.88 **	1.3	−4.13 **	

IBW = initial body weight; DM = dry matter; ME = metabolizable energy; ADG = average daily gain; ADFI = average daily feed intake; G:F = gain-to-feed ratio; N/A = not available. ^1^ Asterisk marks (*,**) represent statistical tendency (*p* < 0.10) and significant difference (*p* < 0.05), respectively. ^2^ The percentage increase or decrease in the ADG, ADFI, and G:F of animals was determined as: [(mean value in the high β-mannans group − mean value in the control group)/mean value in the control group] × 100, and all percentage changes were recalculated in this study based on the reported values from the original studies. ^3^ The dietary contents of both β-mannans and tNSP were determined by multiplying the inclusion rate of each feedstuff by its respective value obtained from Supplementary Table S1 and summing these values. ^4^ The chemical composition was an analyzed value.

## 4. Mechanism of Exogenous β-Mannanase on Intestinal Microbiota, Intestinal Health, Nutrient Utilization, and Growth Performance

Dietary β-mannans have shown negative impacts on monogastric animals, necessitating strategies to maintain intestinal health and growth performance [5,21,49,50,51,54,55,56,57,58,59,60,61]. Dietary β-mannanase supplementation serves as a strategy to mitigate these negative impacts on the diversity and abundance of intestinal microbiota, intestinal health, nutrient utilization, and growth performance of monogastric animals (Figure 1). This enzymatic application expands the traditional focus on merely improving digestion efficiency to address functional roles that target immune stressors on the intestine. In this context, the hydrolysis of β-mannans via β-mannanase generates prebiotic oligosaccharides like MOS, which positively modulate the diversity and abundance of intestinal microbiota and promote SCFA production. Therefore, β-mannanase supplementation serves as a nutritional strategy to reduce digesta viscosity and mitigate its negative impacts on the intestinal microbiota, intestinal health, nutrient utilization, and growth performance of chickens [8,82] and pigs [83].

### 4.1. Modulation of Intestinal Microbiota: Reduced Digesta Viscosity and Oligosaccharides

The effects of exogenous β-mannanase supplementation on the intestinal microbiota and health of chickens and pigs can be explained through two modes of action: the reduction in digesta viscosity and the generation of MOS via the hydrolysis of β-mannans, which have prebiotic effects [12,52,61,84]. Among these modes of action, reducing digesta viscosity is considered one of the primary mechanisms, yet published studies have often reported highly inconsistent results across different studies [52,85,86]. It remains unclear whether this lack of consistency stems from variations in baseline levels of dietary β-mannans or from discrepancies in the efficacy or dose of the β-mannanase used (Table S2). To determine the mitigation effects of β-mannanase on the lnRR of digesta viscosity in the small intestine of chickens fed diets varying in dietary β-mannan contents compared to the group without β-mannanase, meta-analysis was conducted using dietary β-mannans as the independent variable.

lnRR_Digesta viscosity in chicken_ = 0.31 − 0.584 × β-mannans (%; DM basis)

where the slope for β-mannans was −0.584 (standard error = 0.228; 95% confidence interval (CI): −1.05 to −0.12; *p* < 0.05).

To express this relationship in a biologically meaningful way, the lnRR equation was back-transformed into percentage change using ΔDigesta viscosity (%) = (e^0.31−0.584×β-mannans (%)^ − 1) × 100, as described in Section 2.3. This back-transformation indicated that every 1% increase in dietary β-mannan contents (DM basis) was estimated to reduce digesta viscosity in the small intestine by 44.3% (95% CI: −65.0 to −11.3%) in the β-mannanase group compared with the group without β-mannanase (Figure 2). This implies that when chickens fed high β-mannan diets have increased digesta viscosity in the small intestine, β-mannanase supplementation can mitigate this adverse increase in digesta viscosity. The evaluated range of 0.43 to 1.96% β-mannans in the diet (DM basis; Table S2), and the meta-analysis should not be extrapolated beyond this observed range. However, due to the use of different enzyme activity units (U/kg, TMU/kg, or IU/kg) among studies [87,88], the effects of β-mannanase per unit could not be standardized. Total NSP was initially considered as a covariate in the model, as tNSP can be confounding effects on digesta viscosity in the small intestine of animals [21,22,23]. However, β-mannans constitute part of tNSP fractions, which may introduce multicollinearity. Thus, collinearity diagnostics for the digesta viscosity were analyzed and the analysis determined a VIF of 1.15, and a Pearson correlation coefficient of r = 0.36 (*p* < 0.05) between dietary β-mannans and tNSP. The results suggest that including tNSP as a covariate may not distort parameter coefficients in the model. Because the slope for tNSP, however, did not show significance, it was removed from the final meta-analysis model to maintain model parsimony. Thus, the final meta-analysis was structured with dietary β-mannan content as the fixed effect and study as the random effect [29,30]. The random effects of study accounted for around 96% of the total variance in the model for digesta viscosity in the small intestine of chickens (σ^2^ study = 0.164, σ^2^ residual = 0.0067), indicating substantial heterogeneity among studies [31]. As this heterogeneity ratio exceeded the 50% threshold for the digesta viscosity dataset, a sensitivity analysis using influence diagnostics was performed (Table S5). This analysis identified a previous study [51] as exerting disproportionate leverage on the pooled estimate of the slope for β-mannans, which may be attributed to the use of galactomannans as the β-mannan source in that study. Therefore, the estimated dose–response relationship between dietary β-mannan contents and the mitigating effect of β-mannanase on digesta viscosity in the small intestine of chickens should be interpreted with caution. Publication bias for digesta viscosity in the small intestine of chickens was assessed using visual inspection of the funnel plot and Egger’s regression test. The funnel plot did not show marked asymmetry (Figure S1), and Egger’s test did not detect significant small-study effects (intercept = 2.81, *p* = 0.311), indicating there was no evidence of publication bias [33]. In pigs, β-mannanase supplementation has also been shown to decrease digesta viscosity in the small intestine compared to diets without β-mannanase supplementation [12,83,84] (Table S2). However, due to the limited number of studies in pigs, a meta- analysis could not be conducted (Table S2). Collectively, β-mannanase can effectively reduce digesta viscosity in the small intestine, thereby positively influencing the intestinal microbiota, intestinal health, and nutrient utilization of the animals.

In the small intestine, β-mannanase hydrolyzes the β-1,4-mannan backbone, thereby reducing digesta viscosity and generating MOS [89]. These changes create a more favorable intestinal environment by promoting populations of lactate- and SCFA-producing bacteria and limiting the pathogenic colonization of the intestinal mucosa [84,90] (Table 4). A previous study reported that increasing β-mannanase inclusion levels up to 0.03% linearly reduced *E. coli* counts in the ileal and cecal digesta of pigs [84]. A possible reason for this is the prebiotic effect of MOS generated from the hydrolysis of β-mannans by β-mannanase, which may promote populations of beneficial bacteria and limit the ecological niche available for pathogenic colonization; however, the precise mechanism underlying this effect remains to be investigated.

**Table 4:** Metabolites and intestinal microbiota in chickens and pigs fed high β-mannan diets with β-mannanase. Changes were indicated using ↑ (increase) and ↓ (decrease).

Age or IBW	NSP Composition ^1^ (% in DM Diet)		Experimental Period (d)	β-Mannanase (% or Unit/kg) ^2,3^	Result	Reference
	β-Mannans	Total NSP (tNSP)				
Chicken (d)						
1	0.59	12.5	21, 42	300 U/kg	Content: ↑ isobutyrate at 21 d (cecal digesta)	[13]
1	0.60	12.9	21, 42	0.04%	Index: ↑ Chao 1 at 21 d and ↓ chao1 at 42 d; RA: ↑ *Limosilactobacillus reuteri*, ↓ *Streptococcus pasteurians*, ↓ *Romboutsia ilealis* (ileum) at 42 d; ↑ *Anaerobacterium chartisolvens*, ↑ *Alistipes onderdonkii*, ↓ *Intestinimonas butyriciproducens* (cecum) at 42 d	[90]
Pig (kg)						
5.9	0.69	10.8	42	400 U/kg	RA: ↑ *Prevotella*, ↓ *Eubacterium*; Index: ↑ Shannon, ↑ Simpson (cecal digesta)	[91]
6.0	1.97	11.6	42	0 to 0.03%	Count: (linear) ↓ *Escherichia coli* (ileum and cecal digesta)	[84]
6.2	0.53	11.3	37	800 TMU/kg	RA: ↓ Helicobacteraceae, ↑ Streptococcaceae, ↑ Ruminococcaceae (jejunal mucosa)	[5]
6.9	0.46	12.2	35	96,000 U/kg	Count: ↓ *Escherichia coli* (cecal digesta)	[92]
7.5	0.65 ^4^	11.3 ^4^	23	400 U/kg	Content: ↑ butyrate (ileal digesta)	[93]

IBW = initial body weight; DM = dry matter; RA = relative abundance. ^1^ The dietary contents of both β-mannans and tNSP were determined by multiplying the inclusion rate of each feedstuff by its respective value obtained from Supplementary Table S1 and summing these values. ^2^ β-mannanase activity units (U and TMU) represent degradation rate of mannose-containing substrate per minute under specific reaction conditions (e.g., pH, temperature, and substrate sources) [12,94]. ^3^ Direct comparison of β-mannanase inclusion levels across studies was not possible, as β-mannanase activity units (U/kg or TMU/kg) were determined under non-standardized reaction conditions [87,88], and some studies reported inclusion solely as a % without enzyme activity. Thus, direct comparison based on β-mannanase units across studies was not considered in this review. ^4^ The chemical composition was an analyzed value.

In addition to this microbial competition, the reduction in digesta viscosity reduces the opportunistic pathogenic bacteria, creating a highly favorable environment for SCFA-producing bacteria [18,35]. Dietary β-mannanase also positively modulates the diversity and abundance of intestinal microbiota, enhancing the production of SCFA [49,93]. In pigs, β-mannanase supplementation increased the relative abundance (RA) of *Prevotella* in the cecal digesta [91], as well as Ruminococcaceae and Streptococcaceae in the jejunal mucosa [5]. These genera may utilize pyruvate via the acetyl-CoA and Wood-Ljungdahl pathways to generate acetate [95], thereby potentially expanding the luminal acetate pool in the intestine. Furthermore, elevated RA of *Limosilactobacillus reuteri* in the ileum of chickens in response to β-mannanase supplementation can be attributed to an increase in lactate content, which could promote a favorable environment for lactate-producing bacteria [90]. The accumulation of acetate and lactate can act as substrates that promote nutrient substrate sharing among the intestinal microbiota via the butyryl-CoA:acetate CoA-transferase pathway [71]. The coordinated microbial interplay may ultimately account for the increase in butyrate content in the ileal digesta following β-mannanase supplementation [93]. Therefore, changes in the luminal metabolites and the diversity and abundance of intestinal microbiota resulting from β-mannanase supplementation can modulate the mucosa-associated microbiota, which interacts closely with host epithelial cells via PRR to improve intestinal health of monogastric animals [29,96,97]. By suppressing pathogenic colonization and positively modulating the diversity and abundance of mucosa-associated microbiota, β-mannanase supplementation downregulates the relative expression of Toll-like receptor-4 in the small intestine of chickens [8,14] and pigs [57], a key PRR involved in the innate immune response and the detection of lipopolysaccharides from Gram-negative bacteria, reducing immune responses [96]. However, the effects of β-mannanase supplementation on the diversity and abundance of intestinal microbiota have been inconsistent across studies [13], possibly due to variations in substrate availability, enzyme activity, and experimental conditions [98]. Collectively, β-mannanase supplementation possesses a primary mode of action that degrades dietary β-mannans, reducing digesta viscosity and generating beneficial MOS in the small intestine when animals are fed high β-mannan diets. Both effects can be beneficially related to the intestinal microbiota and intestinal health of monogastric animals.

### 4.2. Improvements in Intestinal Barrier Function, Structure, and Mucosal Immunity

As described in Section 4.1, the hydrolysis of dietary β-mannans by β-mannanase in the small intestine reduces digesta viscosity, and generates prebiotic MOS, together exerting beneficial effects on intestinal health, particularly mucosal immunity, as well as intestinal barrier function and structure (Table 5).

The reduction in receptor overstimulation attenuates intestinal immune responses and decreases oxidative damage in the small intestine of monogastric animals [12,99]. In chickens, β-mannanase reduces mucosal inflammation by reducing the mRNA expression of NF-κB and IL-18, concurrently increasing anti-inflammatory cytokines like TGF-β and IL-10 in the ileum [13]. Similarly, in pigs, increasing levels of β-mannanase lead to a linear decrease in pro-inflammatory cytokines, such as TNF-α [12,83], alongside a reduction in systemic inflammatory and stress indicators, including serum white blood cell (WBC), C-reactive protein (CRP), haptoglobin, and cortisol [91]. Furthermore, the mitigation of this immune reaction decreases lipid and protein oxidation within the jejunal mucosal tissue, leading to a linear decline in malondialdehyde (MDA) and protein carbonyl (PC) contents [12]. By reducing these pro-inflammatory and oxidative damage products, β-mannanase supplementation can reduce intestinal epithelial cell damage and the energy and nutrient expenditure required for intestinal repair processes [92,100]. When the epithelium is relieved from continuous, inflammation-driven cellular repair, the available energy and nutrients can be reallocated to reinforce the intestinal structure [70,101,102]. The reduced β-mannan environment in the intestine leads to an increase in tight junction proteins, including claudin-1, ZO-1, and occludin, in the small intestine of chickens [13] and pigs [12]. The fortification of these cellular junctions enhances intestinal barrier function and prevents increased intestinal permeability. The protective effect blocks the leakage of harmful elements into the bloodstream, as shown by the decreased contents of diamine oxidase (DAO) and endotoxins (ET) in the serum of chickens [13]. Ultimately, this continuous protection of the intestinal barrier allows for improved intestinal structure. Across multiple studies, β-mannanase increased VH and the VH:CD in the duodenum, jejunum, and ileum of chickens [6,103], as well as in the jejunum of pigs [12,92]. Collectively, these findings demonstrate that β-mannanase supplementation can enhance overall intestinal health in monogastric animals.

**Table 5:** Immune responses, oxidative damage products, and intestinal integrity and structure in chickens and pigs fed high β-mannan diets with β-mannanase. Changes were indicated using ↑ (increase) and ↓ (decrease).

Age or IBW	NSP Composition ^1^ (% in DM Diet)		Experimental Period (d)	β-Mannanase (% or Unit/kg) ^2,3^	Result	Reference
	β-Mannans	Total NSP (tNSP)				
Immune response and oxidative damage product						
Chicken (d)						
1	0.59	12.5	21, 42	300 U/kg	mRNA expression: ↑ TGF-β3, ↓ NF-κB, ↓ IL-18 at 21 d; ↑ IL-10 at 42 d (ileum)	[13]
1	0.55	12.0	21	10,000 U/kg	Content: ↓ IgM (serum)	[104]
			21	20,000 U/kg	Content: ↓ IgG (serum)	
1	0.61	12.8	42	400 U/kg	Content: ↓ IgA, ↓ IgG, ↓ IgM (serum)	[105]
Pig (kg)						
5.9	0.69	10.8	7, 14	400 U/kg	Content: ↓ WBC, ↓ CRP, ↓ TGF-β1 at d 7; ↓ PCV, ↓ cortisol at d 14 (serum)	[91]
6.2	0.52	11.7	42	800 TMU/kg	Content: ↓ IL-8 (jejunum)	[57]
8.7 ^4^	0.53	12.4	42	800 U/kg	Content: ↓ IgG (serum)	[106]
9.6	1.14	19.2	10	89,600 U/kg	Content: ↓ IL-1α, ↓ haptoglobin (serum)	[107]
15.4	0.72	14.3	10	0 to 600 U/kg	Content: (linear) ↓ TNF-α, ↓ IgG, ↓ MDA, ↓ PC (jejunum); (linear) ↓ TNF-α, ↓ IgG (serum)	[12]
17.6	0.74	14.8	42	400 U/kg	↓ TNF-α (jejunum)	[83]
Intestinal integrity and structure						
Chicken (d)						
1	0.59	12.5	21, 42	300 U/kg	mRNA expression: ↑ claudin-1, ↑ ZO-1 at 21 d; Content: ↓ DAO, ↓ ET at 21 d; Structure: ↓ CD, ↑ VH:CD at 21 d; ↓ CD, ↑ VH:CD at 42 d (jejunum);	[13]
					mRNA expression: ↑ ZO-1 at 21 d; Structure: ↑ VH, ↓ CD, ↑ VH:CD at 21; ↑ VH, ↓ CD, ↑ VH:CD at 42 d (ileum)	
1	0.69	13.7	21	28,000 U/kg	Structure: ↑ VH, ↑ VH:CD (ileum)	[100]
1	0.60	12.7	42	0.05%	No response	[6]
				0.07%	Structure: ↑ VH, ↑ CD, ↑ VH:CD (duodenum); ↑ CD (jejunum and ileum)	
				0.09%	Structure: ↑ VH, ↑ CD, ↑ VH:CD (duodenum); ↑ VH, ↑ CD, ↑ VH:CD (jejunum); ↑ CD (ileum)	
Pig (kg)						
6.9	0.59	11.4	35	96,000 U/kg	Structure: ↑ VH, ↑ VH:CD (jejunum)	[92]
7.5	0.65 ^5^	11.3 ^5^	23	400 U/kg	mRNA expression: ↑ occludin (jejunum)	[93]
15.4	0.72	14.3	10	0 to 600 U/kg	mRNA expression: (tended to linear) ↑ ZO-1 (jejunum); Structure: (linear) ↑ VH, ↑ CD (duodenum); (linear) ↑ VH, ↑ CD, ↓ VH:CD, (linear and quadratic) ↑ crypt cell proliferation (jejunum)	[12]
17.6	0.74	14.7	42	400 U/kg	Structure: ↓ CD, ↑ VH:CD (jejunum)	[83]
35.6	0.63	13.2	13	48,000 U/kg	Structure: ↑ VH, ↑ VH:CD (jejunum)	[108]

IBW = initial body weight; TGF-β3 = transforming growth factor beta 3; NF-κB = nuclear factor kappa-light-chain-enhancer of activated B cells; IL-8 = interleukin-8; IL-18 = interleukin-18; IL-10 = interleukin-10; IgA = immunoglobulin A; IgG = immunoglobulin G; IgM = immunoglobulin M; TNF-α = tumor necrosis factor-alpha; MDA = malondialdehyde; PC = protein carbonyl; IL-1α = interleukin 1-alpha; WBC = white blood cell; CRP = C-reactive protein; TGF-β1 = transforming growth factor beta 1; PCV = packed cell volume; ZO-1 = zonula occludens-1; DAO = diamine oxidase; ET = endotoxin; VH = villus height; CD = crypt depth; VH:CD = villus height-to-crypt depth ratio. ^1^ The dietary contents of both β-mannans and tNSP were determined by multiplying the inclusion rate of each feedstuff by its respective value obtained from Supplementary Table S1 and summing these values. ^2^ β-mannanase activity units (U and TMU) represent degradation rate of mannose-containing substrate per minute under specific reaction conditions (e.g., pH, temperature, and substrate sources) [12,94]. ^3^ Direct comparison of β-mannanase inclusion levels across studies was not possible, as β-mannanase activity units (U/kg or TMU/kg) were determined under non-standardized reaction conditions [87,88], and some studies reported inclusion solely as a % without enzyme activity. Thus, direct comparison based on β-mannanase units across studies was not considered in this review. ^4^ Pigs were fed a mycotoxin-contaminated diet containing 150 µg aflatoxin B1 and 1100 µg deoxynivalenol per kg diet. ^5^ The chemical composition was an analyzed value.

### 4.3. Effects on Nutrient Utilization and Growth Performance

The efficacy of exogenous β-mannanase is highly dependent on the dietary β-mannan content, showing a greater capacity to mitigate the negative impacts on nutrient utilization and growth performance in animals fed high β-mannan diets compared to those on low β-mannan diets [54,109] (Table 6). When β-mannanase is supplemented to high-viscosity diets with high β-mannan contents, it breaks down the β-mannan structure. This action not only reduces digesta viscosity to enhance enzymatic hydrolysis, but also releases nutrients entrapped within the cell wall matrix, leading to increases in ileal digestibility of DM, crude protein (CP), and GE [82,83]. Furthermore, the reduction in digesta viscosity is accompanied by increased endogenous enzyme activities, including trypsin and amylase, which enhance the digestive capacity of animals [100,104]. Previous studies also reported that β-mannanase increased the apparent total tract digestibility of GE and nutrients; this is likely because the enzymatic hydrolysis of β-mannans in the small intestine generates MOS, which subsequently stimulates hindgut fermentation and further contributes to increased total tract digestibility of GE and nutrients in chickens [21] and pigs [84].

**Table 6:** Nutrient utilization in chickens and pigs fed high β-mannan diets with β-mannanase. Changes were indicated using ↑ (increase) and ↓ (decrease).

Age or IBW	NSP Composition ^1^ (% in DM Diet)		Experimental Period (d)	β-Mannanase (% or Unit/kg) ^2,3^	Result	Reference
	β-Mannans	Total NSP (tNSP)				
Chicken (d)						
1	0.59	12.5	21, 42	0.01%	↑ ATTD of DM, GE; ↑ ME	[8]
4	0.49 to 15.24	10.8 to 23.4	10	0.05%	↑ ATTD of DM, CP, EE; ↑ ME	[21]
1	0.60	12.7	21, 42	20,000 U/kg	↑ ME	[104]
1	0.75	14.4	21	400 IU/kg	↑ AID of DM; ↑ ATTD of DM, GE; ↑ ME	[82]
Pig (kg)						
6.0	1.97	11.6	42	0 to 0.03%	(linear) ↑ ATTD of DM, GE, NSP, β-mannans	[84]
6.2	0.52	11.7	42	800 TMU/kg	↑ AID NDF, EE	[57]
7.0	0.59	11.4	35	96,000 U/kg	↑ ATTD of EE	[92]
7.5	0.65 ^4^	11.3 ^4^	23	400 U/kg	↑ AID of β-mannans	[93]
17.6	0.74	14.7	42	400 U/kg	↑ AID of NDF, ADF	[83]
20.6	0.27	8.9	8	800 U/kg	No response	[109]
	0.26	9.6			No response	
	0.32	12.0			↑ ATTD of GE; ↑ ME	
	0.79	15.3			↑ ATTD of GE; ↑ ME	
	0.83	15.8			No response	
	9.28	18.9			(tended to) ↑ ATTD of CP	
	9.46	20.2			↑ ATTD of GE, CP; ↑ ME	
23.6	0.52	13.0	14, 28	0 to 600 U/kg	(linear) ↑ ATTD of DM, CP, NDF, ADF, Ca, P at d 14, 28	[110]
23.5	0.53	14.5	14, 28	400 U/kg	↑ ATTD of DM, GE, CP, NDF, ADF, Ca, P at d 14, 28	
35.6	0.63	13.2	13	48,000 U/kg	↑ ATTD of DM, GE, CP	[108]
34.9	2.52 ^4^	13.8	42	400, 800 U/kg	↑ ATTD of DM, GE, β-mannans	[61]
45.6	0.61 ^4^	12.2	42	400 to 1600 U/kg	(linear) ↑ ATTD of DM, GE, β-mannans	

IBW = initial body weight; DM = dry matter; NSP = non-starch polysaccharide; GE = gross energy; ME = metabolizable energy; CP = crude protein; EE = ether extract; NDF = neutral detergent fiber; ADF = acid detergent fiber; AID = apparent ileal digestibility; ATTD = apparent total tract digestibility. ^1^ The dietary contents of both β-mannans and tNSP were determined by multiplying the inclusion rate of each feedstuff by its respective value obtained from Supplementary Table S1 and summing these values. ^2^ β-mannanase activity units (U, IU, or TMU) represent degradation rate of mannose-containing substrate per minute under specific reaction conditions (e.g., pH, temperature, and substrate sources) [12,94]. ^3^ Direct comparison of β-mannanase inclusion levels across studies was not possible, as β-mannanase activity units (U/kg, IU/kg, or TMU/kg) were determined under non-standardized reaction conditions [87,88], and some studies reported inclusion solely as a % without enzyme activity. Thus, direct comparison based on β-mannanase units across studies was not considered in this review. ^4^ The chemical composition was an analyzed value.

High dietary β-mannan levels inherently reduce growth performance by increasing digesta viscosity, increasing the abundance of opportunistic pathogenic bacteria, stimulating PAMP-like reactions, impairing intestinal health, and reducing ileal and total nutrient digestibility. However, β-mannanase supplementation mitigates these negative impacts through the β-mannan hydrolysis, ultimately improving the growth performance of animals (Tables S3 and S4). However, some previous studies reported that β-mannanase supplementation did not improve growth performance of animals. The lack of detectable improvements in growth performance is likely attributable to low dietary β-mannan levels, a lower proportion of soluble β-mannans, or insufficient enzyme activity.

To determine the effects of β-mannanase on the lnRR of ADG in chickens fed diets with varying in β-mannan contents, a meta-analysis was conducted and provided as below (Figure 3):

lnRR_ADG in chicken_ = −0.0140 + 0.051 × β-mannans (%; DM basis)

where the slope for β-mannans was 0.051 (standard error = 0.014; 95% CI: 0.023 to 0.079; *p* < 0.01). When back-transformed to the percentage change using ΔADG_chicken_ (%) = (e^−0.0140+0.051×β-mannans (%)^ − 1) × 100, this equation indicates that each 1% increase in dietary β-mannan contents (%; DM basis) increases the ADG of chickens by 5.23% (95% CI: 2.33 to 8.22%) with β-mannanase supplementation.

The equation indicates that β-mannanase supplementation was estimated to increase ADG in chickens by 5.23% (*p* < 0.01) for every 1% increase in dietary β-mannan content (%; DM basis) compared to that of chickens fed without β-mannanase (Figure 3). In other words, this implies that when chickens are fed high β-mannan diets, ADG is reduced, and β-mannanase supplementation can mitigate the adverse reduction in growth performance. Specifically, β-mannanase supplementation increases ADG by 5.23% (*p* < 0.01) for every 1% β-mannan content in the diet increase within the evaluated range of 0.30 to 2.15% β-mannans (DM basis), noting that the meta-analysis should not be extrapolated beyond the observed dietary β-mannan range. Lastly, to account for potential collinearity between dietary β-mannans and tNSP, diagnostics were performed, yielding a VIF of 1.14 and a Pearson correlation coefficient of r = 0.35 (*p* < 0.05). Although tNSP did not distort parameter estimates, it was excluded from the final meta-analysis model due to a non-significant slope for tNSP. Accordingly, dietary β-mannan content and study were included in the model as the fixed and random effects, respectively [29,30]. The random effects of study accounted for around 35.8% of the total variance in the model for ADG of chickens (σ^2^ study = 0.00038, σ^2^ residual = 0.00068), indicating moderate heterogeneity among studies [31]. Visual inspection of the funnel plot did not show marked asymmetry (Figure S1), and Egger’s test did not detect significant small-study effects (intercept = 0.431, *p* = 0.310), suggesting there was no evidence of publication bias for the ADG of chickens [33]. This meta-analysis, however, was not able to account for enzyme efficacy and dose-dependent effects of β-mannanase for growth performance, due to non-standardized enzyme activity units across studies; thus, future studies integrating standardized enzyme units and dose-dependent evaluation alongside substrate contents are warranted.

Previous studies also conducted the meta-analysis on the growth performance of chickens [78,103,111]. However, previous studies evaluated only the effect of β-mannanase supplementation itself, without accounting for dietary β-mannan content. This study instead evaluated how the effect of β-mannanase varies with dietary β-mannan content, which provides practical application for nutritionists. In addition, previous studies calculated effect sizes using the standardized mean difference [103] or the mean difference [78], based on the standard error of the mean (SEM) commonly reported in the studies. Because SEM reflects both biological variability and the number of experimental units, studies with a larger number of experimental units can yield disproportionately larger effect sizes, independent of the true treatment effect. To address this, the present study used the lnRR, with weights calculated as *n*/CV^2^, approximating inverse-variance weighting under the assumption of homogeneous variance across treatment and control groups. This approach can allow the number of experimental units to appropriately inform the weighting of each study, whereas the effect size itself is calculated directly from group means and can be readily converted into a percentage change, making the results easy to interpret. Taken together, these differences allow the meta-analysis in this study to provide a practically applicable estimate of β-mannanase efficacy, rather than a single average effect across studies, by accounting for how the effects of the enzyme changes with the β-mannan content in the diet, minimizing the influence of study-to-study differences in sample size on the estimated effect size. This provides nutritionists with a direct and interpretable basis for adjusting β-mannanase supplementation according to β-mannan content in the diet.

Further standardized research is warranted to clarify the efficacy of exogenous β-mannanase in pigs, particularly focusing on specific growth phases. These findings indicate that β-mannanase supplementation is more effective in diets with high β-mannan contents, providing a more economical and practical solution [57] by effectively mitigating the reduction in the growth performance of animals when high β-mannan feedstuffs are used. In the previous studies, β-mannanase supplementation increased ADFI of animals [54,112], potentially because enzymatic degradation of dietary NSP by exogenous enzyme reduced intestinal fill and satiety. The increased ADFI, combined with enhanced nutrient utilization, likely contributed to the observed improvements in ADG of chickens. However, inconsistencies across previous studies (Tables S3 and S4) highlight the need for further investigation to clarify the precise mechanisms underlying ADFI responses. Considering the collective findings on the negative impacts of β-mannans on digesta viscosity, the intestinal microbiota, intestinal health, nutrient utilization, and growth performance of animals, reducing or hydrolyzing β-mannans using β-mannanase could be a valuable dietary strategy for mitigating the negative impacts on chickens and pigs.

### 4.4. Limitations and Future Perspectives

Despite providing comprehensive insights into the effects of dietary β-mannanase in chickens and pigs, several limitations of this review must be acknowledged. First, because not enough data on soluble β-mannan fractions has been reported in the literature, it was difficult to evaluate how the ratio of soluble to total β-mannans affects physiological responses in chickens and pigs. Second, substantial heterogeneity existed among β-mannanases, including differences in microbial origin, thermostability, and non-standardized enzymatic activity units, hindering a standardized dose–response evaluation [87,88]. Consistent with this, influence diagnostics identified a specific study [51] in the digesta viscosity dataset for chickens (Table S5) as exerting disproportionate leverage on the pooled slope of the β-mannans estimate, indicating that the fitted dose–response relationship is not uniformly supported across studies. Notably, a previous study used guar gum [51] as the β-mannan source, a highly soluble galactomannan substrate, suggesting that its disproportionate influence may also reflect the substrate-dependent variation in solubility discussed above in Section 3.2, rather than enzyme-related heterogeneity alone. Because β-mannanase source, dose, and enzymatic activity units varied widely and could not be standardized or included as model covariates, the efficacy of β-mannanase itself likely differed across studies independent of dietary β-mannan content. Accordingly, the observed reduction in digesta viscosity should not be interpreted as being driven by β-mannan content alone; rather, it reflects the combined and potentially overlapping effects of β-mannan structure and β-mannanase efficacy, and the influence of individual studies on this pooled relationship warrants cautious interpretation. Third, variations in specific NSP profiles arising from different feedstuffs could still contribute to response variability [3]. A related limitation concerns measurement uncertainty in the exposure variable itself. Because most primary studies did not report measured β-mannan contents, dietary β-mannan concentrations were largely calculated using literature-derived feedstuff matrix values. Although representative mean values were utilized (Table S1), actual β-mannan contents in feedstuffs can vary depending on cultivar, geographical origin, processing conditions, and analytical methodology [113], introducing potential exposure misclassification into the meta-analysis.

In addition, although study eligibility criteria and precision-based weighting helped mitigate the influence of variable study quality, a formal risk-of-bias scoring tool was not applied; this represents a limitation of the meta-analysis in this study and a direction for future refinement. Finally, from a methodological standpoint, meta-analysis was constrained for certain parameters, particularly in pigs and for specific intestinal health parameters such as diversity and abundance of intestinal microbiota, metabolites, and immune responses, due to the limited number of eligible studies and heterogeneity in reporting metrics, necessitating qualitative synthesis instead. To validate these findings and advance mechanistic understanding, expanded pig dataset evaluations are warranted.

## 5. Conclusions

Dietary β-mannans, particularly those with a high proportion of soluble β-mannans, increase digesta viscosity in the small intestine and disrupt the diversity and the balance of the intestinal microbiota, including an increase in opportunistic pathogenic microbiota and a concurrent reduction in SCFA-related metabolites. Dietary β-mannans may stimulate unnecessary innate immune responses and increase oxidative damage in the small intestine due to structural similarity between β-mannans and microbial PAMPs. The increased digesta viscosity and immunological alterations induced by β-mannans subsequently impair overall intestinal health, including disruption of small intestinal barrier function and structure, and reduced nutrient utilization, which can lead to reduced growth performance in chickens and pigs. Dietary β-mannanase supplementation, however, has been proposed to mitigate the immune reaction by preventing PAMP-like overstimulation, thereby preserving energy and nutrients that would otherwise be diverted toward intestinal immune responses (such as inflammation) and epithelial tissue repair. Dietary β-mannanase reduces digesta viscosity and increases the production of MOS in the small intestine, positively modulating the diversity and abundance of intestinal microbiota, improving intestinal health, and enhancing nutrient utilization. Together, the combination of enhanced intestinal health and increased nutrient utilization improves the growth performance of monogastric animals.

The beneficial effects of β-mannanase are pronounced in diets containing high levels of dietary β-mannans. Specifically, as the inclusion of cost-effective feedstuffs such as SBM and guar meal increases dietary β-mannan content, β-mannanase supplementation becomes increasingly effective in mitigating the negative impacts of β-mannans on intestinal health and growth performance of chickens. Dietary β-mannanase supplementation was indicated by meta-analysis to mitigate the negative impacts of β-mannans; specifically, it was estimated to reduce digesta viscosity in the small intestine by an average of 44.3% (95% CI: −65.0 to −11.3%) and to increase ADG by an average of 5.23% (95% CI: 2.33 to 8.22%) when β-mannan contents increase by 1% in the diet (evaluation range: 0.30 to 2.15% β-mannans; DM basis) relative to chickens fed diets without the enzyme. This indicates that β-mannanase supplementation can effectively mitigate the negative impacts of β-mannans in feedstuffs on intestinal health and growth of chickens, allowing increased use of β-mannan-rich feedstuffs. Although the meta-analysis could not be conducted for pigs due to a limited number of studies and datasets, the underlying mode of action of β-mannanase is generally considered consistent between chickens and pigs. Collectively, this review provides valuable mechanistic insights suggesting that β-mannanase supplementation may facilitate greater inclusion of high β-mannan feedstuff by reducing these negative impacts on intestinal microbiota, intestinal health, nutrient utilization, and growth performance of monogastric animals. However, these model-derived values from the meta-analysis are limited by variations in enzyme efficacy, non-standardized activity units, variable dosages across studies, and the absence of a formal risk-of-bias assessment. In particular, the estimated reduction in digesta viscosity by β-mannanase should be interpreted with caution given the substantial heterogeneity and disproportionate influence of individual studies identified through sensitivity analysis. This highlights the need for further research, particularly in pigs, to clarify β-mannanase efficacy when used in diets with different types of dietary β-mannans.

## Figures and Tables

**Figure 1:**
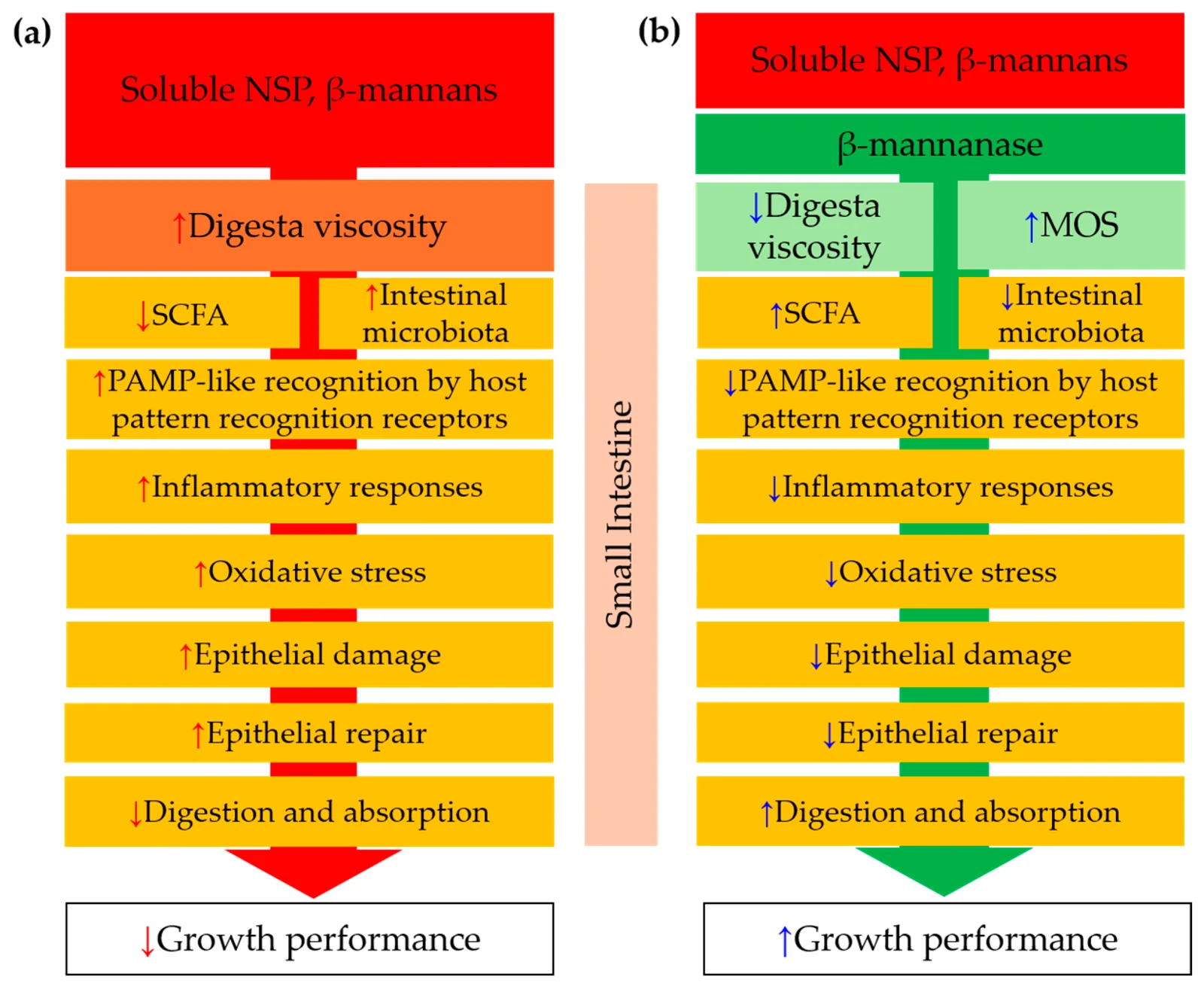
Proposed pathways of (**a**) Impacts of soluble non-starch polysaccharide (NSP) fractions, including β-mannans on the intestinal microbiota, intestinal health, nutrient utilization, and growth performance of monogastric animals (chickens and pigs), and (**b**) Dietary strategies using β-mannanase to mitigate the negative impacts of soluble NSP fractions, including β-mannans on the intestinal microbiota, intestinal health, nutrient utilization, and growth performance of chickens and pigs. ↑ and ↓ indicate the proposed increase and decrease, respectively.

**Figure 2:**
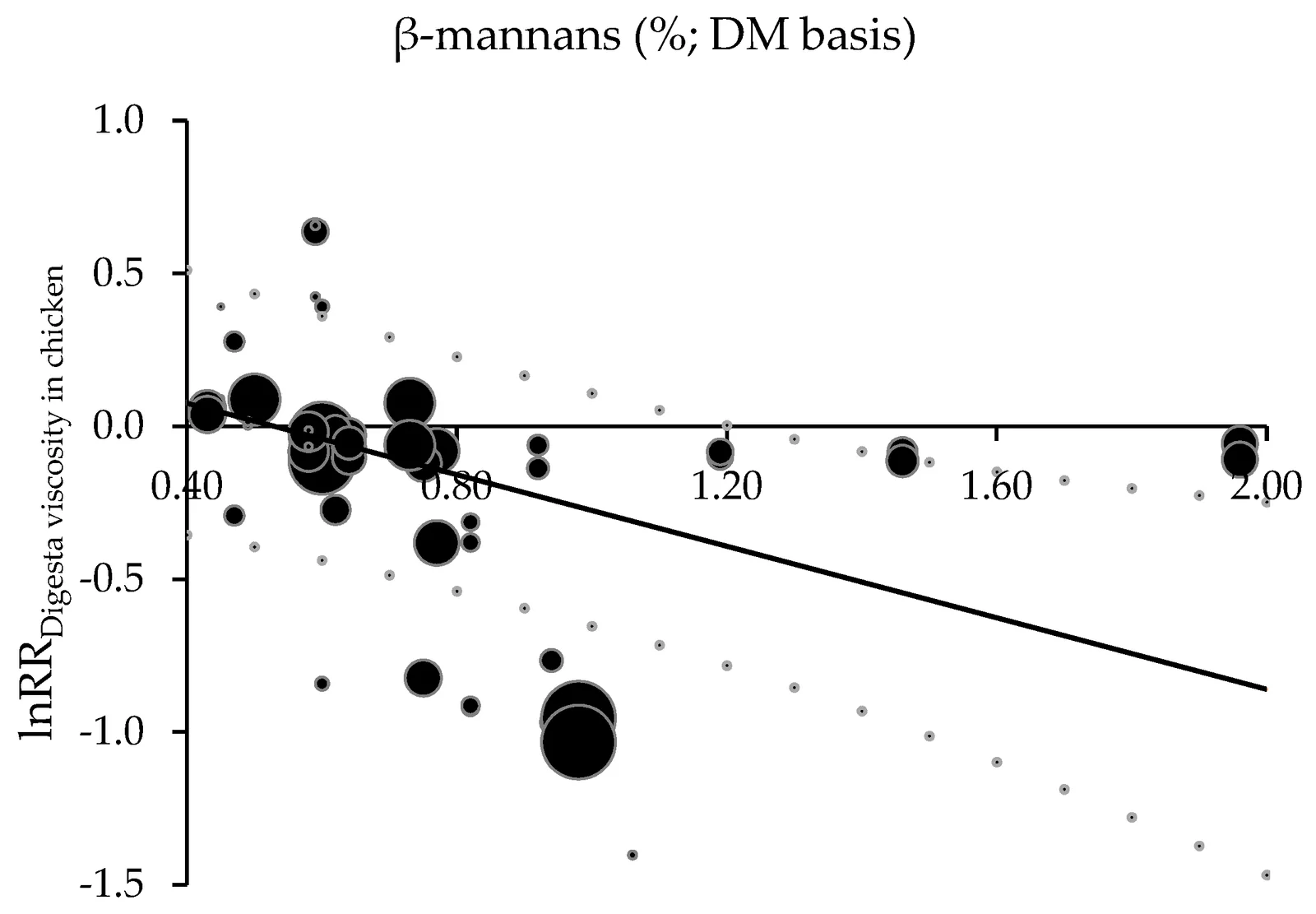
The log response ratio (lnRR) of digesta viscosity in the small intestine of chickens fed diets with varying β-mannan contents and β-mannanase supplementation, relative to the group without β-mannanase. The meta-analysis was conducted using the PROC MIXED procedure with 54 observations based on 8 studies from 8 peer-reviewed papers to determine the effects of β-mannanase supplementation on the lnRR of digesta viscosity in the small intestine of chickens (lnRR_Digesta viscosity of chicken_). The equation for chickens fed diets with β-mannanase compared to without β-mannanase (solid line, ●) was as follows: lnRR_Digesta viscosity of chicken_ = 0.31 − 0.584 × β-mannans (%; DM basis), R^2^ = 0.17, where the slope for β-mannans was −0.584 (standard error = 0.228; 95% confidence interval: −1.05 to −0.12; *p* < 0.05). When back-transformed to the percentage change using ΔDigesta viscosity in chicken (%) = (e^0.31−0.584×β-mannans (%)^ − 1) × 100, this equation indicated that each 1% increase in dietary β-mannan contents (DM basis) was estimated to reduce digesta viscosity in the small intestine of chickens by 44.3% (95% confidence interval: −65.0 to −11.3%) with β-mannanase supplementation. The gray dotted lines represent the 95% confidence interval of the regression line. Dietary β-mannan composition is expressed on a DM basis. The solid regression line reflects this relationship between dietary β-mannans and the β-mannanase-induced change in digesta viscosity in the small intestine of chickens. In the figure, each data point represents the observed lnRR of digesta viscosity between chickens fed a diet with β-mannanase supplementation and those fed the corresponding control diet, with both diets having the same dietary β-mannans. The dietary β-mannan contents ranged from 0.43 to 1.96% (DM basis). The bubble size represents the weight of each observation, which was determined as the sample size (*n*) divided by the squared coefficient of variation (*n*/CV^2^) [26]. The dietary β-mannanase level was not considered due to the use of different enzyme units among the studies.

**Figure 3:**
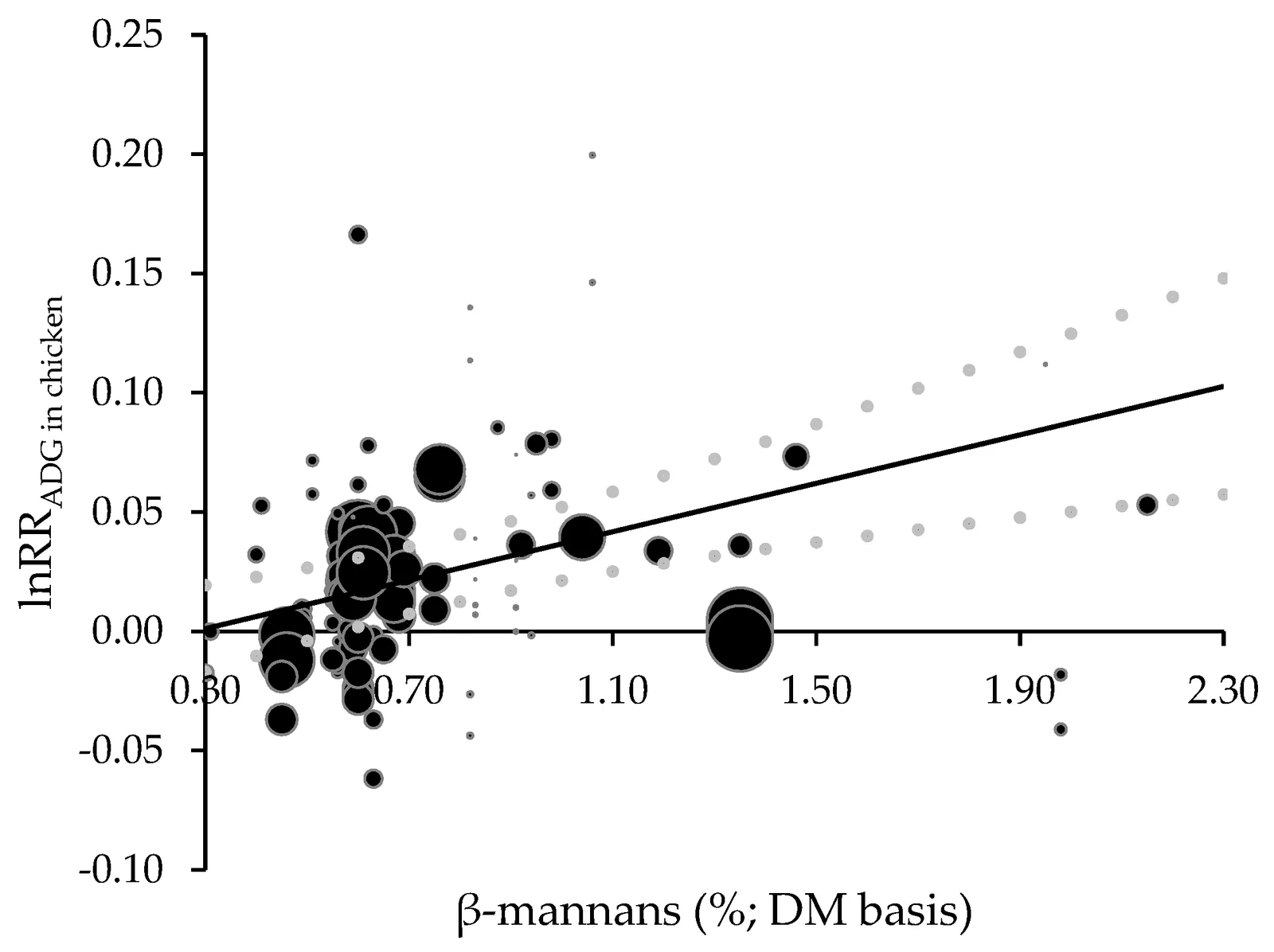
The log response ratio (lnRR) of average daily gain (ADG) in chickens fed diets with varying β-mannan contents and β-mannanase supplementation, relative to the group without β-mannanase. The meta-analysis was conducted using the PROC MIXED procedure with 102 observations based on 30 studies from 28 peer-reviewed papers for chickens to determine the effects of β-mannanase supplementation on the lnRR of ADG in chickens (lnRR_ADG in chicken_). The equation for chickens fed diets with β-mannanase compared to those without β-mannanase (solid line, ●) was as follows: lnRR_ADG in chicken_ = −0.0140 + 0.051 × β-mannans (%; DM basis), R^2^ = 0.24, where the slope for β-mannans was 0.051 (standard error = 0.014; 95% confidence interval: 0.023 to 0.079; *p* < 0.01). When back-transformed to the percentage change using ΔADG_chicken_ (%) = (e^−0.014+0.051×β-mannans (%)^ − 1) × 100, the equation indicated that each 1% increase in dietary β-mannan contents (%; DM basis) was estimated to increase ADG in chickens by 5.23% (95% confidence interval: 2.33 to 8.22%) with β-mannanase supplementation. The gray dotted lines represent the 95% confidence interval of the regression line. Dietary β-mannans are expressed on a DM basis. In the figure, each data point represents the observed lnRR of ADG between chickens fed a diet with β-mannanase supplementation and those fed the corresponding control diet, with both diets having the same dietary β-mannans. The dietary β-mannans ranged from 0.30 to 2.15% in chicken diets (DM basis). The bubble size represents the weight of each observation, which was calculated as the sample size (*n*) divided by the squared coefficient of variation (*n*/CV^2^) [26]. The dietary β-mannanase level was not considered due to the use of different enzyme units among the studies.

## Data Availability

The data presented in this study are available on request from the corresponding author.

## Supplementary Materials

The following supporting information can be downloaded at: https://www.mdpi.com/article/10.3390/ani16193036/s1, Table S1: Contents of total non-starch polysaccharide (NSP), total β-mannans, and soluble β-mannans in feedstuffs for monogastric animals; Table S2: Digesta viscosity in chickens and pigs fed high β-mannan diets with β-mannanase; Table S3: Growth performance in chickens fed high β-mannan diets with β-mannanase; Table S4: Growth performance in pigs fed high β-mannan diets with β-mannanase; Table S5: Study-level influence diagnostics for the meta- analysis of digesta viscosity in the small intestine of chickens; Figure S1: Funnel plots for assessing publication bias in the meta-analysis of (a) digesta viscosity in the small intestine and (b) average daily gain (ADG) of chickens. References [114,115,116,117,118,119,120,121,122,123,124,125,126,127,128,129,130,131,132,133,134,135,136,137,138,139] are cited in the Supplementary Materials.

## Abbreviations

The following abbreviations are used in this manuscript:

AA	Amino acid
ADF	Acid detergent fiber
ADFI	Average daily feed intake
ADG	Average daily gain
AID	Apparent ileal digestibility
AME	Apparent metabolizable energy
ANC	Anti-nutritional compound
ATTD	Apparent total tract digestibility
CCLi2	K203 macrophage chemokine
CD	Crypt depth
CI	Confidence interval
CP	Crude protein
CRP	C-reactive protein
DAO	Diamine oxidase
DM	Dry matter
EE	Ether extract
FITC	Fluorescein isothiocyanate
ET	Endotoxins
G:F	Gain-to-feed ratio
GE	Gross energy
IBW	Initial body weight
ICC	Intraclass correlation coefficient
IgA	Immunoglobulin A
IgG	Immunoglobulin G
IgM	Immunoglobulin M
IL-1	Interleukin-1
IL-6	Interleukin-6
IL-10	Interleukin-10
IL-18	Interleukin-18
M:G	Mannose-to-galactose ratio
MDA	Malondialdehyde
ME	Metabolizable energy
MOS	Manno-oligosaccharides
NDF	Neutral detergent fiber
NF-κB	Nuclear factor kappa-light-chain-enhancer of activated B cells
NSP	Non-starch polysaccharide
PAMP	Pathogen-associated molecular pattern
PC	Protein carbonyl
PCV	Packed cell volume
PICOS	Population, intervention, comparator, outcome, and study design
PKM	Palm kernel meal
PRR	Pattern recognition receptor
r	Pearson correlation coefficient
RA	Relative abundance
lnRR	Log response ratio
SAA	Serum amyloid A
SBM	Soybean meal
SCFA	Short-chain fatty acids
SEM	Standard error of the mean
SID	Standardized ileal digestibility
TMU	Thermostable mannanse unit
tNSP	Total non-starch polysaccharide
TGF-β1	Transforming growth factor beta 1
TGF-β3	Transforming growth factor beta 3
TNF-α	Tumor necrosis factor-alpha
VIF	Variance inflation factor
VH	Villus height
VH:CD	Villus height-to-crypt depth ratio
WBC	White blood cell
ZO-1	Zonula occludens-1
α-1GP	Alpha-1-acid glycoprotein

## References

[1] Knudsen K.E.B. (2001). The nutritional significance of “dietary fibre” analysis. Anim. Feed Sci. Technol..

[2] Kocher A., Choct M., Ross G., Broz J., Chung T.K. (2003). Effects of enzyme combinations on apparent metabolizable energy of corn-soybean meal-based diets in broilers. J. Appl. Poult. Res..

[3] Knudsen K.E.B. (1997). Carbohydrate and lignin contents of plant materials used in animal feeding. Anim. Feed Sci. Technol..

[4] Woyengo T.A., Beltranena E., Zijlstra R.T. (2014). Nonruminant nutrition symposium: Controlling feed cost by including alternative ingredients into pig diets: A review. J. Anim. Sci..

[5] Baker J.T., Deng Z., Sokale A., Frederick B., Kim S.W. (2024). Nutritional and functional roles of β-mannanase on intestinal health and growth of newly weaned pigs fed two different types of feeds. J. Anim. Sci..

[6] Mehri M., Adibmoradi M., Samie A., Shivazad M. (2010). Effects of β-mannanase on broiler performance, gut morphology and immune system. Afr. J. Biotechnol..

[7] Arsenault R.J., Lee J.T., Latham R., Carter B., Kogut M.H. (2017). Changes in immune and metabolic gut response in broilers fed β-mannanase in β-mannan-containing diets. Poult. Sci..

[8] Zhang X., Wang B., Ban Z., Wang M., Wang Y., Wang X., Guo Y. (2025). Impact of mannanase on broiler performance, intestinal health, and energy utilization with varying soybean meal levels. Anim. Adv..

[9] Embgenbroich M., van der Zande H.J., Hussaarts L., Schulte-Schrepping J., Pelgrom L.R., García-Tardón N., Schlautmann L., Stoetzel I., Händler K., Lambooij J.M. (2021). Soluble mannose receptor induces proinflammatory macrophage activation and metaflammation. Proc. Natl. Acad. Sci. USA.

[10] Taylor M.E., Drickamer K. (1993). Structural requirements for high affinity binding of complex ligands by the macrophage mannose receptor. J. Biol. Chem..

[11] Abreu M.T., Vora P., Faure E., Thomas L.S., Arnold E.T., Arditi M. (2001). Decreased expression of Toll-like receptor-4 and MD-2 correlates with intestinal epithelial cell protection against dysregulated proinflammatory gene expression in response to bacterial lipopolysaccharide. J. Immunol..

[12] Jang K.B., Kim Y.I., Duarte M.E., Kim S.W. (2024). Effects of β-mannanase supplementation on intestinal health and growth of nursery pigs. J. Anim. Sci..

[13] Zhang X., Xu H., Gong L., Wang J., Fu J., Lv Z., Zhou L., Li X., Liu Q., Xia P. (2024). Mannanase improves the growth performance of broilers by alleviating inflammation of the intestinal epithelium and improving intestinal microbiota. Anim. Nutr..

[14] Liu H., Liu W., Ai M., Hao X., Zhang Q., Ren J., Zhang K. (2024). Effects of β-mannanase supplementation on productive performance, inflammation, energy metabolism, and cecum microbiota composition of laying hens fed with reduced-energy diets. Poult. Sci..

[15] JøRgensen H., Zhao X.-Q., Knudsen K.E.B., Eggum B.O. (1996). The influence of dietary fibre source and level on the development of the gastrointestinal tract, digestibility and energy metabolism in broiler chickens. Br. J. Nutr..

[16] Moran E.T., Bedford M.R. (2022). Large intestinal dynamics differ between fowl and swine: Anatomical modifications, microbial collaboration, and digestive advantages from fibrolytic enzymes. Anim. Nutr..

[17] Liberati A., Altman D.G., Tetzlaff J., Mulrow C., Gøtzsche P.C., Ioannidis J.P., Clarke M., Devereaux P.J., Kleijnen J., Moher D. (2009). The PRISMA statement for reporting systematic reviews and meta-analyses of studies that evaluate health care interventions: Explanation and elaboration. Ann. Intern. Med..

[18] Choi H., Duarte Y.G., Pasquali G.A., Kim S.W. (2024). Investigation of the nutritional and functional roles of a combinational use of xylanase and β-glucanase on intestinal health and growth of nursery pigs. J. Anim. Sci. Biotechnol..

[19] Choct M., Hughes R.J., Wang J., Bedford M., Morgan A., Annison G. (1996). Increased small intestinal fermentation is partly responsible for the anti-nutritive activity of non-starch polysaccharides in chickens. Br. Poult. Sci..

[20] Hung Y.-T., Zhu J., Shurson G.C., Urriola P.E., Saqui-Salces M. (2022). Decreased nutrient digestibility due to viscosity is independent of the amount of dietary fibre fed to growing pigs. Br. J. Nutr..

[21] Sundu B., Kumar A., Dingle J. (2006). Response of broiler chicks fed increasing levels of copra meal and enzymes. Int. J. Poult. Sci..

[22] Choct M. (1997). Feed non-starch polysaccharides: Chemical structures and nutritional significance. Feed Milling Int..

[23] Knudsen K.E.B. (2014). Fiber and nonstarch polysaccharide content and variation in common crops used in broiler diets. Poult. Sci..

[24] Thompson S.G., Higgins J.P. (2002). How should meta-regression analyses be undertaken and interpreted?. Stat. Med..

[25] Marín-Martínez F., Sánchez-Meca J. (2010). Weighting by inverse variance or by sample size in random-effects meta-analysis. Educ. Psychol. Meas..

[26] Nakagawa S., Noble D.W., Lagisz M., Spake R., Viechtbauer W., Senior A.M. (2023). A robust and readily implementable method for the meta-analysis of response ratios with and without missing standard deviations. Ecol. Lett..

[27] Dormann C.F., Elith J., Bacher S., Buchmann C., Carl G., Carré G., Marquéz J.R.G., Gruber B., Lafourcade B., Leitão P.J. (2013). Collinearity: A review of methods to deal with it and a simulation study evaluating their performance. Ecography.

[28] Rocha G.C., Duarte M.E., Kim S.W. (2022). Advances, implications, and limitations of low-crude-protein diets in pig production. Animals.

[29] Kim S.W., Duarte M.E., Gormley A.R. (2026). Research summary: Influence of immune and oxidative stress biomarkers ins the jejunal mucosa on growth performance of nursery pigs. Anim. Front..

[30] Choi H., Sung J.Y., Son J., Kim S.W. (2026). Recent updates on amino acid requirements of pigs based on phenotypic improvements: Invited review. Anim. Biosci..

[31] Higgins J.P.T., Thompson S.G., Deeks J.J., Altman D.G. (2003). Measuring inconsistency in meta-analyses. Br. Med. J..

[32] Christensen R., Pearson L.M., Johnson W. (1992). Case-deletion diagnostics for mixed models. Technometrics.

[33] Egger M., Smith G.D., Schneider M., Minder C. (1997). Bias in meta-analysis detected by a simple, graphical test. Br. Med. J..

[34] Knudsen K.E.B., Hedemann M.S., Lærke H.N. (2012). The role of carbohydrates in intestinal health of pigs. Anim. Feed Sci. Technol..

[35] Hopwood D.E., Pethick D.W., Pluske J.R., Hampson D.J. (2004). Addition of pearl barley to a rice-based diet for newly weaned piglets increases the viscosity of the intestinal contents, reduces starch digestibility and exacerbates post-weaning colibacillosis. Br. J. Nutr..

[36] Bjergegaard C., Sørensen H., Sørensen S. (1997). Dietary fibres-important parts of high quality food and feeds. J. Anim. Feed Sci..

[37] Choi H., Sung J.Y., Kim B.G. (2020). Neutral detergent fiber rather than other dietary fiber types as an independent variable increases the accuracy of prediction equation for digestible energy in feeds for growing pigs. Asian-Australas. J. Anim. Sci..

[38] Theander O., Westerlund E., Åman P., Graham H. (1989). Plant cell walls and monogastric diets. Anim. Feed Sci. Technol..

[39] Zhang L., Tizard I.R. (1996). Activation of a mouse macrophage cell line by acemannan: The major carbohydrate fraction from Aloe vera gel. Immunopharmacol..

[40] Dos Santos V.R.F., Souza B.W.S., Teixeira J.A., Vicente A.A., Cerqueira M.A. (2015). Relationship between galactomannan structure and physicochemical properties of films produced thereof. J. Food Sci. Technol..

[41] Baker J.T., Duarte M.E., Holanda D.M., Kim S.W. (2021). Friend or foe? Impacts of dietary xylans, xylooligosaccharides, and xylanases on intestinal health and growth performance of monogastric animals. Animals.

[42] Chen T., Chen D., Tian G., Zheng P., Mao X., Yu J., He J., Huang Z., Luo Y., Luo J. (2020). Effects of soluble and insoluble dietary fiber supplementation on growth performance, nutrient digestibility, intestinal microbe and barrier function in weaning piglet. Anim. Feed Sci. Technol..

[43] Knudsen K.B., Hansen I. (1991). Gastrointestinal implications in pigs of wheat and oat fractions: 1. Digestibility and bulking properties of polysaccharides and other major constituents. Br. J. Nutr..

[44] Duarte M.E., Zhou F.X., Dutra W.M., Kim S.W. (2019). Dietary supplementation of xylanase and protease on growth performance, digesta viscosity, nutrient digestibility, immune and oxidative stress status, and gut health of newly weaned pigs. Anim. Nutr..

[45] Smits C.H.M., Veldman A., Verstegen M.W.A., Beynen A.C. (1997). Dietary carboxymethylcellulose with high instead of low viscosity reduces macronutrient digestion in broiler chickens. J. Nutr..

[46] Lee G.I., Hedemann M.S., Jørgensen H., Knudsen K.E.B. (2022). Influence of dietary fibre on nutrient digestibility and energy utilisation in growing pigs fed diets varying in soluble and insoluble fibres from co-products. Animal.

[47] Liu Q., Zhang W.M., Zhang Z.J., Zhang Y.J., Zhang Y.W., Chen L., Zhuang S. (2016). Effect of fiber source and enzyme addition on the apparent digestibility of nutrients and physicochemical properties of digesta in cannulated growing pigs. Anim. Feed Sci. Technol..

[48] Bartelt J., Jadamus A., Wiese F., Swiech E., Buraczewska L., Simon O. (2002). Apparent precaecal digestibility of nutrients and level of endogenous nitrogen in digesta of the small intestine of growing pigs as affected by various digesta viscosities. Arch. Anim. Nutr..

[49] Van Nevel C.J., Decuypere J.A., Dierick N.A., Molly K. (2005). Incorporation of galactomannans in the diet of newly weaned piglets: Effect on bacteriological and some morphological characteristics of the small intestine. Arch. Anim. Nutr..

[50] Owusu-Asiedu A.J.F.J., Patience J.F., Laarveld B., Van Kessel A.G., Simmins P.H., Zijlstra R.T. (2006). Effects of guar gum and cellulose on digesta passage rate, ileal microbial populations, energy and protein digestibility, and performance of grower pigs. J. Anim. Sci..

[51] Caldas J.V., Vignale K., Boonsinchai N., Wang J., Putsakum M., England J.A., Coon C.N. (2018). The effect of β-mannanase on nutrient utilization and blood parameters in chicks fed diets containing soybean meal and guar gum. Poult. Sci..

[52] Lee J.T., Bailey C.A., Cartwright A.L. (2003). β-mannanase ameliorates viscosity-associated depression of growth in broiler chickens fed guar germ and hull fractions. Poult. Sci..

[53] Saittagaroon S., Kawakishi S., Namiki M. (1983). Characterisation of polysaccharides of copra meal. J. Sci. Food Agric..

[54] Gharaei M.A., Dastar B., Nameghi A.H., Tabar G.H., Shargh M.S. (2012). Effects of guar meal with and without β-mannanas enzyme on performance and immune response of broiler chicks. Int. Res. J. Appl. Basic Sci..

[55] Latham R.E., Williams M.P., Walters H.G., Carter B., Lee J.T. (2018). Efficacy of β-mannanase on broiler growth performance and energy utilization in the presence of increasing dietary galactomannan. Poult. Sci..

[56] Mellouk A., Mahmood T., Jlali M., Vieco-Saiz N., Michel V., Cozannet P., Ozbek S., Mercier Y., Devillard E., Consuegra J. (2024). Potential of guar gum as a leaky gut model in broilers: Digestibility, performance, and microbiota responses. Anim. Nutr..

[57] Choi H., Deng Z., Sokale A., Von Heimendahl E., Amerah A.M., Kim S.W. (2026). Investigation of nutritional and functional roles of β-mannanase on intestinal health and growth performance of nursery pigs fed low-cost formulated diets with high phytase inclusion. J. Anim. Sci..

[58] El-Masry K.N., Ragaa N.M., Tony M.A., El-Banna R.A. (2017). Effect of dietary inclusion of guar meal with or without β-mannanase supplementation on broiler performance and immunity. Pak. J. Nutr..

[59] Daskiran M., Teeter R.G., Fodge D., Hsiao H.Y. (2004). An evaluation of endo-β-D-mannanase (Hemicell) effects on broiler performance and energy use in diets varying in β-mannan content. Poult. Sci..

[60] Kim H.J., Nam S.O., Jeong J.H., Fang L.H., Yoo H.B., Yoo S.H., Hong J.S., Son S.W., Ha S.H., Kim Y.Y. (2017). Various levels of copra meal supplementation with β-Mannanase on growth performance, blood profile, nutrient digestibility, pork quality and economical analysis in growing-finishing pigs. J. Anim. Sci. Technol..

[61] Kim J.S., Ingale S.L., Hosseindoust A.R., Lee S.H., Lee J.H., Chae B.J. (2017). Effects of mannan level and β-mannanase supplementation on growth performance, apparent total tract digestibility and blood metabolites of growing pigs. Animal.

[62] Kim J.C., Hansen C.F., Mullan B.P., Pluske J.R. (2012). Nutrition and pathology of weaner pigs: Nutritional strategies to support barrier function in the gastrointestinal tract. Anim. Feed Sci. Technol..

[63] Daneshmand A., Kermanshahi H., Mohammed J., Sekhavati M.H., Javadmanesh A., Ahmadian M., Alizadeh M., Razmyar J., Kulkarni R.R. (2022). Intestinal changes and immune responses during *Clostridium perfringens*-induced necrotic enteritis in broiler chickens. Poult. Sci..

[64] Moran E.T. (2014). Intestinal events and nutritional dynamics predispose *Clostridium perfringens* virulence in broilers. Poult. Sci..

[65] Wellock I.J., Fortomaris P.D., Houdijk J.G.M., Wiseman J., Kyriazakis I. (2008). The consequences of non-starch polysaccharide solubility and inclusion level on the health and performance of weaned pigs challenged with enterotoxigenic *Escherichia coli*. Br. J. Nutr..

[66] Gerritsen R., van Der Aar P., Molist F. (2012). Insoluble nonstarch polysaccharides in diets for weaned piglets. J. Anim. Sci..

[67] Hermes R.G., Molist F., Ywazaki M., Nofrarías M., Gomez de Segura A., Gasa J., Pérez J.F. (2009). Effect of dietary level of protein and fiber on the productive performance and health status of piglets. J. Anim. Sci..

[68] Molist F., de Segura A.G., Pérez J.F., Bhandari S.K., Krause D.O., Nyachoti C.M. (2010). Effect of wheat bran on the health and performance of weaned pigs challenged with *Escherichia coli* K88+. Livest. Sci..

[69] Choct M., Kocher A., Waters D.L.E., Pettersson D., Ross G. (2004). A comparison of three xylanases on the nutritive value of two wheats for broiler chickens. Br. J. Nutr..

[70] Choi H., Deng Z., Kim S.W. (2025). Impacts of different dietary soybean meal levels on jejunal immunity of nursery pigs at different days post-weaning. J. Anim. Sci. Biotechnol..

[71] Duncan C.J., Pugh N., Pasco D.S., Ross S.A. (2002). Isolation of a galactomannan that enhances macrophage activation from the edible fungus *Morchella esculenta*. J. Agric. Food Chem..

[72] Vendele I., Willment J.A., Silva L.M., Palma A.S., Chai W., Liu Y., Feizi T., Spyrou M., Stappers M.H., Brown G.D. (2020). Mannan detecting C-type lectin receptor probes recognise immune epitopes with diverse chemical, spatial and phylogenetic heterogeneity in fungal cell walls. PLoS Pathog..

[73] Kuipers S., Aerts P.C., van Dijk H. (2003). Differential microorganism-induced mannose-binding lectin activation. FEMS Immunol. Med. Microbiol..

[74] Sukhithasri V., Nisha N., Biswas L., Kumar V.A., Biswas R. (2013). Innate immune recognition of microbial cell wall components and microbial strategies to evade such recognitions. Microbiol. Res..

[75] Kéry V., Křepinský J.J., Warren C.D., Capek P., Stahl P.D. (1992). Ligand recognition by purified human mannose receptor. Arch. Biochem. Biophys..

[76] Fernández N., Alonso S., Valera I., Vigo A.G.l., Renedo M., Barbolla L., Crespo M.S. (2005). Mannose-containing molecular patterns are strong inducers of cyclooxygenase-2 expression and prostaglandin E2 production in human macrophages. J. Immunol..

[77] Chen W.L., Chen H.L., Guo G.w., Huang Y.C., Chen C.Y., Tsai Y., Huang K.F., Yang C.H. (2018). Locust bean gum galactomannan hydrolyzed by thermostable β-d-mannanase may reduce the secretion of pro-inflammatory factors and the release of granule constituents. Int. J. Biol. Macromol..

[78] Kiarie E.G., Steelman S., Martinez M., Livingston K. (2021). Significance of single β-mannanase supplementation on performance and energy utilization in broiler chickens, laying hens, turkeys, sows, and nursery-finish pigs: A meta-analysis and systematic review. Transl. Anim. Sci..

[79] Peng S.Y., Norman J., Curtin G., Corrier D., McDaniel H.R., Busbee D. (1991). Decreased mortality of Norman murine sarcoma in mice treated with the immunomodulator, Acemannan. Mol. Biother..

[80] Huntley N.F., Gould S.A., Patience J.F. (2019). Evaluation of the effect of β-mannanase supplementation and mannans on nursery pig growth performance and serum acute-phase protein concentrations. Can. J. Anim. Sci..

[81] Seo J., Kim W., Kim J., Kim J.K., Kim S.C., Jang Y., Jang K., Kim K., Kim B., Park S. (2015). Effects of palm kernel expellers on growth performance, nutrient digestibility, and blood profiles of weaned pigs. Asian-Australas J. Anim. Sci..

[82] Kong C., Lee J.H., Adeola O. (2011). Supplementation of β-mannanase to starter and grower diets for broilers. Can. J. Anim. Sci..

[83] Chen H., Garavito-Duarte Y., Kim Y.I., Zhang S., Kim S.W. (2025). Effects of β-mannanase on intestinal health and growth performance of nursery to growing pigs fed diets with DDGS. J. Anim. Sci..

[84] Tajudeen H., Mun J.Y., Ha S., Hosseindoust A., Kinara E., Lokhande A., Ingale S.L., Kim J.S. (2025). The immunomodulatory activities of a thermostable galacto-mannanase and their impact on the performance of weaned piglets. Anim. Feed Sci. Technol..

[85] Hosseindoust A., Lee S., Nho W.G., Song Y.H., Shin J.S., Ingale S.L., Rathi P.C., Choi J., Chae B., Kim J. (2019). A dose-response study to evaluate the effects of pH-stable β-mannanase derived from *Trichoderma citrinoviride* on growth performance, nutrient retention, and intestine morphology in broiler chickens. Ital. J. Anim. Sci..

[86] Barekatain R., Hall L., Chrystal P.V., Fickler A. (2024). Nutrient utilisation and growth performance of broiler chickens fed standard or moderately reduced dietary protein diets with and without β-mannanase supplementation. Anim. Nutr..

[87] Adeola O., Cowieson A.J. (2011). Board-invited review: Opportunities and challenges in using exogenous enzymes to improve nonruminant animal production. J. Anim. Sci..

[88] Choct M. (2006). Enzymes for the feed industry: Past, present and future. World’s Poult. Sci. J..

[89] Suryawanshi R.K., Kango N. (2021). Production of mannooligosaccharides from various mannans and evaluation of their prebiotic potential. Food Chem..

[90] Bortoluzzi C., Scapini L.B., Ribeiro M.V., Pivetta M.R., Buzim R., Fernandes J.I.M. (2019). Effects of β-mannanase supplementation on the intestinal microbiota composition of broiler chickens challenged with a coccidiosis vaccine. Livest. Sci..

[91] Jang K.B., Kim Y., Ahn J., Lee J.I., Park S., Choe J., Kim Y., Lee J.H., Kyoung H., Song M. (2024). Dietary β-mannanase reduced post-weaning diarrhea of pigs by positively modulating gut microbiota and attenuating systemic immune responses. Anim. Microbiom..

[92] Jang J.-C., Kim K.H., Jang Y.D., Kim Y.Y. (2020). Effects of dietary β-mannanase supplementation on growth performance, apparent total tract digestibility, intestinal integrity, and immune responses in weaning pigs. Animals.

[93] Tiwari U.P., Chen H., Kim S.W., Jha R. (2018). Supplemental effect of xylanase and mannanase on nutrient digestibility and gut health of nursery pigs studied using both in vivo and in vitro models. Anim. Feed Sci. Technol..

[94] Williams M.P., Brown B., Rao S., Lee J.T. (2014). Evaluation of beta-mannanase and nonstarch polysaccharide-degrading enzyme inclusion separately or intermittently in reduced energy diets fed to male broilers on performance parameters and carcass yield. J. Appl. Poult. Res..

[95] Koh A., De Vadder F., Kovatcheva-Datchary P., Bäckhed F. (2016). From dietary fiber to host physiology: Short-chain fatty acids as key bacterial metabolites. Cell.

[96] Gormley A.R., Duarte M.E., Deng Z., Kim S.W. (2024). *Saccharomyces* yeast postbiotics mitigate mucosal damages from F18+ *Escherichia coli* challenges by positively balancing the mucosal microbiota in the jejunum of young pigs. Anim. Microbiome.

[97] Lopez-Rincon M.A., Gormley A.R., Kim S.W. (2026). Microbiota–host–immune interactions in nursery pigs: Dietary composition and its impact on intestinal microbiota and mucosal immunity for intestinal health. Anim. Nutr..

[98] Merkesvik J., Lindstad L.J., Umu Ö.C.O., Sandholm R.M., La Rosa S.L., Hvidsten T.R., Pope P.B., Westereng B. (2026). In vitro model reveals structural and metabolic insights into the porcine cecal microbiota in response to β-mannan exposure. Appl. Environ. Microbiol..

[99] Zhang L., Huan H., Zhang K., Tu Y., Yan J., Zhang H., Xi Y. (2024). The effects of β-mannanase supplementation on growth performance, digestive enzyme activity, cecal microbial communities, and short-chain fatty acid production in broiler chickens fed diets with different metabolizable energy levels. J. Anim. Sci..

[100] Zou J., Zheng P., Zhang K., Ding X., Bai S. (2013). Effects of exogenous enzymes and dietary energy on performance and digestive physiology of broilers. J. Anim. Sci. Biotechnol..

[101] Tóth Š., Fagová Z., Holodová M., Čurgali K., Mechírová E., Kunová A., Maretta M., Nemcová R., Gancarčíková S., Danková M. (2025). Intestinal mucosal turnover in germ-free piglets infected with *E. coli*. J. Mol. Histol..

[102] Duarte M.E., Kim S.W. (2022). Significance of mucosa-associated microbiota and its impacts on intestinal health of pigs challenged with F18+ *E. coli*. Pathogens.

[103] Kim H.W., Kwon C.H., Lee J.H., Kang M.S., Kil D.Y. (2024). Effect of dietary β-mannanase supplementation on growth performance, intestinal morphology, digesta viscosity, and nutrient utilization in broiler chickens: Meta-analysis and meta-regression. Anim. Biosci..

[104] Li Y., Chen X., Chen Y., Li Z., Cao Y. (2010). Effects of β-mannanase expressed by *Pichia pastoris* in corn-soybean meal diets on broiler performance, nutrient digestibility, energy utilization and immunoglobulin levels. Anim. Feed Sci. Technol..

[105] Ferreira H.C., Hannas M.I., Albino L.F.T., Rostagno H.S., Neme R., Faria B.D., Xavier M.L., Rennó L.N. (2016). Effect of the addition of β-mannanase on the performance, metabolizable energy, amino acid digestibility coefficients, and immune functions of broilers fed different nutritional levels. Poult. Sci..

[106] Garavito-Duarte Y.R., Son J., Weaver A.C., Kim S.W. (2025). Functional efficacies of humate and β-mannanase against Aflatoxin B1 and Deoxynivalenol in the diets for nursery pigs. Toxins.

[107] Huntley N.F., Nyachoti C.M., Patience J.F. (2018). Lipopolysaccharide immune stimulation but not β-mannanase supplementation affects maintenance energy requirements in young weaned pigs. J. Anim. Sci. Biotechnol..

[108] Galli G.M., Andretta I., Carvalho C.L., Stefanello T.B., Souza de Lima Cony B., Zem Fraga A., Ludwig Takeuti K., da Rosa A.B., Kipper M. (2024). Effects of β-mannanase alone or combined with multi-carbohydrase complex in corn-soybean meal diets on nutrient metabolism and gut health of growing pigs. Animals.

[109] Jang K.B., Zhao Y., Kim Y.I., Pasquetti T., Kim S.W. (2023). Effects of bacterial β-mannanase on apparent total tract digestibility of nutrients in various feedstuffs fed to growing pigs. Anim. Biosci..

[110] Lv J.N., Chen Y.Q., Guo X.J., Piao X.S., Cao Y.H., Dong B. (2013). Effects of supplementation of β-mannanase in corn-soybean meal diets on performance and nutrient digestibility in growing pigs. Asian-Australas. J. Anim. Sci..

[111] Kipper M., Andretta I., Quadros V.R.d., Schroeder B., Pires P.G.d.S., Franceschina C.S., Hickmann F.M.W., França I. (2020). Performance responses of broilers and pigs fed diets with β-mannanase. Rev. Bras. Zootec..

[112] Mussini F.J., Coto C.A., Goodgame S.D., Lu C., Karimi A.J., Lee J.H., Waldroup P.W. (2011). Effect of ß-mannanase on broiler performance and dry matter output using corn-soybean meal based diets. Int. J. Poult. Sci..

[113] Ibáñez M.A., De Blas C., Cámara L., Mateos G.G. (2020). Chemical composition, protein quality and nutritive value of commercial soybean meals produced from beans from different countries: A meta-analytical study. Anim. Feed Sci. Technol..

[114] Jaworski N.W., Lærke H.N., Bach Knudsen K.E., Stein H.H. (2015). Carbohydrate composition and in vitro digestibility of dry matter and nonstarch polysaccharides in corn, sorghum, and wheat and coproducts from these grains. J. Anim. Sci..

[115] Gutierrez N.A., Serão N.V.L., Kerr B.J., Zijlstra R.T., Patience J.F. (2014). Relationships among dietary fiber components and the digestibility of energy, dietary fiber, and amino acids and energy content of nine corn coproducts fed to growing pigs. J. Anim. Sci..

[116] Rodehutscord M., Rückert C., Maurer H.P., Schenkel H., Schipprack W., Bach Knudsen K.E., Schollenberger M., Laux M., Eklund M., Siegert W. (2016). Variation in chemical composition and physical characteristics of cereal grains from different genotypes. Arch. Anim. Nutr..

[117] Slominski B.A., Campbell L.D., Guenter W. (1994). Carbohydrates and dietary fiber components of yellow-and brown-seeded canola. J. Agric. Food Chem..

[118] Lee E.C., Kim K.H., Mugeniwayesu C., Jeong Y.J., Kil D.Y. (2025). Effects of dietary β-mannanase supplementation in low-energy and low-protein diets containing high-mannan ingredients on growth performance, meat quality, intestinal morphology, and liver health in broiler chickens. Anim. Biosci..

[119] Azarfar A. (2013). Effect of hemicell enzyme on the performance, growth parameter, some blood factors and ileal digestibility of broiler chickens fed corn/soybean-based diets. J. Cell Anim. Biol..

[120] Jackson M.E., Geronian K., Knox A., McNab J., McCartney E. (2004). A dose-response study with the feed enzyme beta-mannanase in broilers provided with corn-soybean meal based diets in the absence of antibiotic growth promoters. Poult. Sci..

[121] Zou X.T., Qiao X.J., Xu Z.R. (2006). Effect of β-mannanase (Hemicell) on growth performance and immunity of broilers. Poult. Sci..

[122] Latham R.E., Williams M., Smith K., Stringfellow K., Clemente S., Brister R., Lee J.T. (2016). Effect of β-mannanase inclusion on growth performance, ileal digestible energy, and intestinal viscosity of male broilers fed a reduced-energy diet. J. Appl. Poult. Res..

[123] Marçal B.V., Silva R.L., Maria L.P., dos Santos Araújo R., Dourado L.R.B., da Silva M.K., Sakomura N.K. (2025). Effects of beta-mannanase supplementation on growth performance, digestibility, intestinal morphometry, and cecal microbiota in broiler chickens. Livest. Sci..

[124] Yaqoob M.U., Yousaf M., Khan M.I., Wang M. (2022). Effect of β-mannanase supplementation on growth performance, ileal digestibility, carcass traits, intestinal morphology, and meat quality in broilers fed low-ME diets. Animals.

[125] Ahsan T., Tahir M., Khan R.U., Ahmad S., Khan N.A., Konca Y., Alhidary I.A., Abdelrahman S., Tarricone S. (2024). Impact of varying levels of soy hulls and β-mannanase enzyme supplementation on growth performance, carcass characteristics, nutrient utilization and blood biochemical profile in broiler chickens. Ital. J. Anim. Sci..

[126] Chegeni A., Torki M., Kamyab A. (2011). Effects of β-mannanase-based enzyme in corn-soy and corn-soy-canola diets on broiler performance. J. Appl. Anim. Res..

[127] Hashim M.M., El-Safty S.A., El-Eraqi K.G., El-Sherif H.M.R., Azza T., Marwa K., Ibrahim H.M., Abdelhady A.Y.M. (2020). Effect of β-mannanase enzymes supplementation to energy deficient diets on productive performance, physiological and carcass traits of broilers. Int. J. Poult. Sci..

[128] Kim E., Choct M., Fickler A., Pasquali G.A.M., Hall L., Crowley T.M., Sharma N.K. (2025). Supplementation of β-mannanase alone or in combination with xylanase and β-glucanase enhanced growth performance, non-starch polysaccharide degradation, and gastrointestinal environment of broilers offered wheat-based diets. Anim. Nutr..

[129] Chuppava B., Abd El-Wahab A., Schiel B., Ratert C., Reckels B., Visscher C., Kamphues J., Lingens J.B. (2022). Impacts of mannanase supplementation in guar meal by-product on broiler chickens performance, foot pad health and selected organ traits: A pilot study. Eur. Poult. Sci..

[130] de Souza M., Eeckhaut V., Goossens E., Ducatelle R., Van Nieuwerburgh F., Poulsen K., Baptista A.A.S., Bracarense A.P.F.R.L., Van Immerseel F. (2023). Guar gum as galactomannan source induces dysbiosis and reduces performance in broiler chickens and dietary β-mannanase restores the gut homeostasis. Poult. Sci..

[131] Carr S.N., Allee G.L., Rincker P.J., Fry R.S., Boler D.D. (2014). Effects of endo-1, 4-β-d-mannanase enzyme (Hemicell HT 1.5×) on the growth performance of nursery pigs. Prof. Anim. Sci..

[132] Acosta J.P., Lee S.A., Fickler A., Stein H.H. (2025). Tolerance of weanling pigs and effects on growth performance of supplementing corn-soybean meal-based diets with graded levels of a novel exogenous β-mannanase. Transl. Anim. Sci..

[133] Jones C.K., Bergstrom J.R., Tokach M.D., DeRouchey J.M., Goodband R.D., Nelssen J.L., Dritz S.S. (2010). Efficacy of commercial enzymes in diets containing various concentrations and sources of dried distillers grains with solubles for nursery pigs. J. Anim. Sci..

[134] Pettey L.A., Carter S.D., Senne B.W., Shriver J.A. (2002). Effects of beta-mannanase addition to corn-soybean meal diets on growth performance, carcass traits, and nutrient digestibility of weanling and growing-finishing pigs. J. Anim. Sci..

[135] Jacela J.Y., Dritz S.S., DeRouchey J.M., Tokach M.D., Goodband R.D., Nelssen J.L. (2010). Effects of supplemental enzymes in diets containing distillers dried grains with solubles on finishing pig growth performance. Prof. Anim. Sci..

[136] Kim J.S., Ingale S.L., Lee S.H., Kim K.H., Lee J.H., Chae B.J. (2013). Effects of energy levels of diet and β-mannanase supplementation on growth performance, apparent total tract digestibility and blood metabolites in growing pigs. Anim. Feed Sci. Technol..

[137] Jeon S.M., Hosseindoust A., Choi Y.H., Kim M.J., Kim K.Y., Lee J.H., Kil D.Y., Kim B.G., Chae B.J. (2019). Comparative standardized ileal amino acid digestibility and metabolizable energy contents of main feed ingredients for growing pigs when adding dietary β-mannanase. Anim. Nutr..

[138] Liu F., Li J., Ni H., Azad M.A.K., Mo K., Yin Y. (2023). The effects of phytase and non-starch polysaccharide-hydrolyzing enzymes on trace element deposition, intestinal morphology, and cecal microbiota of growing–finishing pigs. Animals.

[139] Yoon S.Y., Yang Y.X., Shinde P.L., Choi J.Y., Kim J.S., Kim Y.W., Yun K., Jo J.K., Lee J.H., Ohh S.J. (2010). Effects of mannanase and distillers dried grain with solubles on growth performance, nutrient digestibility, and carcass characteristics of grower-finisher pigs. J. Anim. Sci..

