# Supplementary material for: Impact of Dietary β-Mannans on the Intestinal-Nutritional Interface and Growth Performance of Chickens and Pigs: Challenges and Strategies via Exogenous β-Mannanase Based on Meta-Analysis
Source: Animals (Basel). 2026 Sep 26;16(19):3036. doi: 10.3390/ani16193036 (PMC13663395; doi:10.3390/ani16193036)
Supplement: Supplementary file 1 [file animals-16-03036-s001.zip › animals-4535841-supplementary.pdf]

Supplementary Table S1. Contents of total non-starch polysaccharide (NSP), total β-mannans, and soluble β-mannans in feedstuffs for monogastric animals (DM basis).

Item	Total NSP	Total β-mannans	Soluble β-mannans	Reference
Cereal grains and co-products				
Corn	9.44 (SD = 1.61; n = 5)	0.28 (SD = 0.10; n = 4)	0.18 (SD = 0.10; n = 4)	[1-3]
Corn DDGS	22.70 (SD = 3.25; n = 2)	1.40 (SD = 0.71; n = 2)	0.50 (SD = 0.10; n = 6)	[2,4]
Corn bran	37.40 (SD = 3.03; n = 4)	0.40 (SD = 0.00; n = 4)	0.07 (SD = 0.06; n = 3)	[1,2,4]
Corn gluten meal	4.25 (SD = 0.92; n = 2)	0.20 (SD = 0.14; n = 2)	0.05 (SD = 0.07; n = 2)	[2,4]
Corn gluten feed	31.90 (SD = 4.53; n = 2)	0.80 (SD = 0.57; n = 2)	0.35 (SD = 0.49; n = 2)	[1,2]
Wheat	10.24 (SD = 5.30; n = 27)	0.25 (SD = 0.05; n = 26)	0.09 (SD = 0.07; n = 26)	[1-3,5]
Wheat bran	36.68 (SD = 6.05; n = 4)	0.78 (SD = 0.10; n = 4)	0.10 (SD = 0.08; n = 4)	[1-3,5]
Wheat middlings	24.85 (SD = 8.27; n = 2)	0.55 (SD = 0.35; n = 2)	0.10 (SD = 0.14; n = 2)	[1,2]
Barley	15.14 (SD = 4.96; n = 14)	0.38 (SD = 0.03; n = 14)	0.11 (SD = 0.02; n = 14)	[2,5]
Soybean hulls	64.50 (n = 1)	5.00 (n = 1)	2.1 (n = 1)	[6]
Protein supplements				
Soybean meal	21.57 (SD = 3.25; n = 7)	1.31 (SD = 0.14; n = 7)	0.50 (SD = 0.10; n = 6)	[1,3]
Canola meal	20.87 (SD = 1.42; n = 22)	1.85 (SD = 0.27; n = 22)	0.10 (SD = 0.10; n = 1)	[1,7]
Corn germ meal	39.45 (SD = 7.00; n = 2)	0.35 (SD = 0.07; n = 2)	<0.01 (n = 2)	[2,4]
Palm kernel meal	46.60 (n = 1)	30.90 (n = 1)	N/A	[1]
Copra meal	42.20 (n = 1)	30.30 (SD = 0.42; n =2)	1.20 (n = 1)	[1,8]
Guar meal	20.40 (n = 1)	10.40 (n = 1)	N/A	[9]
Others				
Guar gum	80.00 (n = 1)	80.00 (n = 1)	N/A	[10]

DM = dry matter; SD = standard deviation; DDGS = distiller dried grains with solubles; N/A = not available.

Supplementary Table S2. Digesta viscosity in chickens and pigs fed high β-mannan diets with β-mannanase [1, 2].

Age or IBW	NSP composition[3] (% in DM diet)		Experimental period (d)	Number of replicates	β-mannanase (% or unit/kg)[4, 5]	Digesta viscosity (% change)	Reference
	β-mannans	Total NSP (tNSP)					
Chicken (d)							
1	0.59	12.7	21	6	109,000 U/kg	−2.2% (ileum)	[11]
					436,000 U/kg	+89.1% **	
	0.82	12.2			109,000 U/kg	−26.9% **	
					436,000 U/kg	−60.1% **	
	0.94	12.0			109,000 U/kg	−53.5% **	
					436,000 U/kg	−62.0% **	
	0.59	12.7			109,000 U/kg	+92.9% **	
					436,000 U/kg	+52.8% **	
	0.82	12.7			109,000 U/kg	−59.9% **	
					436,000 U/kg	−31.6% **	
	1.06	12.8			109,000 U/kg	−75.4% **	
					436,000 U/kg	−81.6% **	
4	0.49 to 15.24	10.8 to 23.4	10	4	0.05%	−9.1% ** (jejunum)	[12]
1	0.60	12.7	42	4	0.05%	−2.8%	[13]
					0.07%	−8.0% **	
					0.09%	−10.8% **	
1	0.65	13.4	21	5	0.05%	−2.4% (ileum)	[14]
	0.92	13.3				−6.0%	
	1.19	13.2				−9.5%	
	1.46	13.1				−8.1%	
	0.65	13.4	42		0.05%	−1.3%	
	0.92	13.3				−12.8%	
	1.19	13.2				−8.0%	
	1.46	13.1				−10.7%	
1	0.64	13.0	35	10	1200 IU/kg	−2.8% (ileum)	[15]
					2400 IU/kg	−6.7%	
					4800 IU/kg	−9.7%	
1	0.47	10.8	14	12	35,000 U/kg	32.0% ** (ileum)	[16]
					70,000 U/kg	−25.3%	
	0.62	11.0			35,000 U/kg	−23.9% **	
					70,000 U/kg	−1.2%	
	0.77	11.1			35,000 U/kg	−7.7% **	
					70,000 U/kg	−31.7% **	
	0.45	10.6	28		35,000 U/kg	+9.6% (ileum)	
					70,000 U/kg	48.0%	
	0.60	10.7			35,000 U/kg	−56.9%	
					70,000 U/kg	48.0%	
	0.75	10.9			35,000 U/kg	−11.4% **	
					70,000 U/kg	−56.1% **	
	0.43	10.5	42		35,000 U/kg	6.0%	

					70,000 U/kg	4.0%	
	0.58	10.7			35,000 U/kg	−7.9%	
					70,000 U/kg	−1.7%	
	0.73	10.8			35,000 U/kg	7.9%	
					70,000 U/kg	−6.0%	
1	0.64	13.9	35	10	800 TMU/kg	−5.3% ** (ileum)	[17]
	0.50	12.5			800 TMU/kg	+9.2% **	
1	0.49	11.2	14	8	29,000 U/kg	+0.4% (jejunum)	[10]
					58,000 U/kg	1.6%	
	0.58	12.5			29,000 U/kg	−1.3%	
					58,000 U/kg	−6.4%	
	0.98	12.9			29,000 U/kg	−61.5% **	
					58,000 U/kg	−64.4% **	
1	1.96	12.2	35	6	400 U/kg	−5.5% (jejunum)	[18]
					800 U/kg	−10.3% **	
Pig (kg)							
6.0 [6]	1.97	11.6	42	10	0.01%	−8.0% (ileum)	[19]
					0.02%	−22.6%	
					0.03%	−29.5%	
6.2	0.53	11.3	37	8	800 TMU/kg	−23.1% ** (jejunum)	[20]
7.5	0.65 [7]	11.3 [7]	20	8	400 U/kg	+5.5% (jejunum)	[21]
15.4 [6]	0.66	13.6	10	12	400 U/kg	−16.4% (jejunum)	[22]
					600 U/kg	−16.0%	
15.7 [6]	0.72	14.3	11	24	200 U/kg	−15.5%	
					400 U/kg	−26.8%	
					600 U/kg	−16.3%	
17.6	0.74	14.7	42	14	400 U/kg	−21.8% ** (jejunum)	[23]

IBW = initial body weight; DM = dry matter. [1] Asterisk marks (*, **) represent statistical tendency ($p < 0.10$) and significant difference ($p < 0.05$), respectively. [2] The percentage increase or decrease of digesta viscosity in the small intestine was determined as: [(mean value in the β-mannanase group – mean value in the control group without β-mannanase)/mean value in the control group without β-mannanase] × 100, and all percentage changes were recalculated in this study based on the reported values from the original studies. [3] The dietary contents of both β-mannans and tNSP were determined by multiplying the inclusion rate of each feedstuff by its respective value obtained from supplementary Table 1 and summing these values. [4] β-mannanase activity units (U and TMU) represent degradation rate of mannose-containing substrate per minute under specific reaction conditions (e.g., pH, temperature, and substrate sources) [22,24]. [5] Direct comparison of β-mannanase inclusion levels across studies was not possible, as β-mannanase activity units (U/kg or TMU/kg) were determined under non-standardized reaction conditions [25,26], and some studies reported inclusion solely as a % without enzyme activity. Thus, direct comparison based on β-mannanase units across studies was not considered in this review. [6] Increasing levels of β-mannanase linearly decreased ($p < 0.05$) the digesta viscosity in the small intestine. [7] The chemical composition was an analyzed value.

Supplementary Table S3. Growth performance in chickens fed high β-mannan diets with β-mannase [1, 2].

Age (d)	NSP composition[3] (% in DM diet)		Experimental period (d)	ME (kcal/kg)	Number of replicates	β-mannanase (% or unit/kg)[4, 5]	Growth performance (% change)			Reference
	β-mannans	Total NSP (tNSP)					ADG	ADFI	G:F	
1	0.31	8.02	21	2950	8	0.01%	0.00	0.00	0.00	[27]
	0.41	9.8					5.41	1.85	3.49	
	0.62	13.0					8.11 **	1.85	6.14 **	
	0.30	8.1	42	3025		0.01%	−1.72	-1.92	0.20	
	0.40	9.6					3.28	0.00	3.28	
	0.60	12.6					6.35	1.96	4.3	
1	0.68	13.6	42	3000	10	28,000 U/kg	0.64	−1.04 *	1.69 **	[28]
1	0.60	12.7	42	2855	5	10,000 U/kg	2.13 **	−2.25 *	4.48 **	[29]
						20,000 U/kg	4.26 **	−2.25 *	6.65 **	
1	0.60	12.7	42	3000	4	80,000 U/kg	−2.34	−2.21	−0.14	[13]
						11,200 U/kg	−2.47	−0.80	−1.68 **	
						14,400 U/kg	−2.80	−3.86	1.10	
2	0.75	14.4	42	3098	8	400 IU/kg	1.04	0.76	0.28	[30]
1	0.56	12.2	42	3025	5	80,000 U/kg	−0.42	0.67	−1.08	[31]
						160,000 U/kg	2.74	3.20	−0.44	
1	0.61	12.8	42	3050	10	400 IU/kg	2.52 **	0.87 *	1.63 **	[32]
1	0.59	12.8	42	2975	15	50,000 U/kg	−0.71	−2.78	2.13	[33]
						80,000 U/kg	4.08 **	−0.12 *	4.21 **	
						110,000 U/kg	4.44 **	−1.12	5.62 **	
19	0.56	12.2	14	3020	6	200 IU/kg	5.08	5.45**	−0.35	[34]
						400 IU/kg	−1.69	0.16	−1.85	
						800 IU/kg	−1.51	1.47	−2.93	
1	0.68	13.6	42	2990	3	41,250 U/kg	2.86 **	0.01	2.84 **	[35]
						82,500 U/kg	4.64 **	−1.06	5.77 **	
						123,750 U/kg	3.18 **	1.76	1.40	
1	0.60	12.8	47	3007	9	57,930 U/kg	18.10 *	14.70	2.96 **	[24]
1	0.62	13.0	17	3015	15	363 U/kg	4.15	1.62	2.49	[36]
	0.58	12.9	42	3051			2.20	0.86	1.32	
1	0.66	13.4	21	2997	10	48,000 IU/kg	−1.01	0.00	−1.01	[37]
1	0.61	12.9	42	3068			1.83	0.90	0.92	
7	0.67	12.9	14	3070	5	32,000 U/kg	1.67	−1.01	2.72	[38]
				3040		64,000 U/kg	3.21	2.64	0.56	
				3010		96,000 U/kg	1.26	−0.20	1.46	
	0.63	12.6	28	3120		32,000 U/kg	-5.98	−5.24	−0.78	
				3090		64,000 U/kg	-0.15	−2.56	2.47	
				3060		96,000 U/kg	-3.62	−4.96	1.41	
1	0.57	12.2	21	3000	6	1000 IU/kg	3.21	−2.21	5.54	[39]
				2930			2.01	0.85	1.15	
				2860			2.39	−1.36	3.8	
	0.55	11.9	42	3000		1000 IU/kg	0.36	−3.22	3.7	
				2930			1.71	1.22	0.49	

				2860	8		1.33	−1.59	2.97	
7	0.49	11.2	14	3000	8	29,000 U/kg	0.95	1.34	−0.39	[10]
						58,000 U/kg	0.58	−0.15	0.74	
	0.58	12.5				29,000 U/kg	−0.11	−0.65	0.54	
						58,000 U/kg	0.11	−6.27	6.8	
	0.98	12.9				29,000 U/kg	6.10	−3.97	10.48	
						58,000 U/kg	8.39	−3.16	11.92	
1	0.83	15.2	21	3000	4	80,000 U/kg	0.71	−1.39	2.13	[40]
						160,000 U/kg	1.12	−0.46	1.59	
	0.91	16.0				80,000 U/kg	0.00	1.08	−1.07	
						160,000 U/kg	1.01	−0.62	1.64	
	0.83	15.2	35			80,000 U/kg	2.21	1.25	0.95	
						160,000 U/kg	3.97	−3.74	8.01	
	0.91	16.0				80,000 U/kg	3.01	−3.91	7.21	
						160,000 U/kg	7.69	−3.61	11.73	
1	0.69	13.8	21	2850	9	66,000 U/kg	2.66	0.95	1.69	[41]
	0.65	13.4	42	2900			5.45 **	0.12	5.32 **	
1	0.51	11.6	35	3020	6	62,500 U/kg	5.93 **	−5.68 **	12.31 **	[42]
						47,400 U/kg	7.43 **	−6.02 **	14.31 **	
1	0.58	14.9	35	3086	8	800 TMU/kg	1.47 *	−5.58	7.46 **	[43]
1	0.59	12.7	21	3075	6	109,000 U/kg	−0.60	3.60	−4.05	[11]
						436,000 U/kg	0.60	3.44	−2.74	
	0.82	12.2				109,000 U/kg	−2.60	0.07	−2.67	
						436,000 U/kg	−4.27	−0.34	−3.95	
	0.94	12.0				109,000 U/kg	−0.16	−6.48	6.76	
						436,000 U/kg	5.87	3.86	1.94	
1	0.59	12.7	20	3075	6	109,000 U/kg	3.60	0.80	2.78 **	
						436,000 U/kg	4.91	1.37	3.50 **	
	0.82	12.7				109,000 U/kg	14.54	11.51	2.72 **	
						436,000 U/kg	12.03	9.80	2.03 **	
	1.06	12.8				109,000 U/kg	15.75	8.12	7.05 **	
						436,000 U/kg	22.09	9.66	11.33 **	
7	0.87[6]	14.0	26	3490	3	171,123 U/kg	8.92	7.07	1.73 **	[44]
1	1.95	13.7	35	3030	12	52,800 U/kg	11.85	3.52	8.05	[45]
1	0.59	12.4	35	3100	4	48,000 U/kg	1.39	−0.13	1.52	[46]
	1.04	12.3					4.03**	−2.92	7.16	
8	0.65	13.4	34	3000	5	0.05%	−0.74	−2.02	1.32	[14]
	0.92	13.3					3.70	1.42	2.25	
	1.19	13.2					3.44	0.46	2.97 *	
	1.46	13.1					7.61 *	5.70 *	1.8	
1	0.46	10.7	28	3168	12	35,000 U/kg	−0.14	−0.27	0.13	[16]
	0.61	10.9					3.36	1.00	2.34	
	0.76	11.0					6.72 **	1.75 **	4.89 **	
	0.46	10.7				70,000 U/kg	−1.19	0.18	−1.37	
	0.61	10.9					2.49	−0.51	3.01	
	0.76	11.0					7.03 **	2.49 **	4.42 *	
1	0.45	10.7	42	3168		35,000 U/kg	−3.62	−1.92	−1.73	

	0.60	10.8					−0.25	−0.80	0.56	
	0.75	11.0					2.25 **	−0.82	3.09 **	
	0.45	10.7				70,000 U/kg	−1.87	−0.92	−0.96	
	0.60	10.8					−1.71	−2.37	0.67	
	0.75	11.0					0.93	−1.12	2.08 *	
1	0.55	11.9	14	3150	5	80,000 U/kg	−1.17	−4.48	3.47	[47]
	0.95	12.3					8.19	4.26	3.77	
	1.35	12.7					3.67	1.62	2.01	
	2.15	13.5					5.45	−2.43	8.08	
1	1.35	12.7	14	3150	6	80,000 U/kg	0.12	−2.37	2.54	
						160,000 U/kg	0.46	−2.16	2.68	
						240,000 U/kg	−0.29	−5.43	5.43	
1	1.98	12.6	35	3050	6	400 U/kg	−4.02 **	0.00	−0.86 **	[18]
						800 U/kg	−1.79 **	0.00	−0.27 **	

IBW = initial body weight; DM = dry matter; ME = metabolizable energy; ADG = average daily gain; ADFI = average daily feed intake; G:F = gain to feed ratio. [1] Asterisk marks (*, **) represent statistical tendency ($p < 0.10$) and significant difference ($p < 0.05$), respectively. [2] The percentage change of the ADG, ADFI, and G:F was determined as: [(mean value in the β-mannanase group – mean value in the control group without β-mannanase)/mean value in the control group without β-mannanase] × 100, and all percentage changes were recalculated in this study based on the reported values from the original studies. [3] The dietary contents of both β-mannans and tNSP were determined by multiplying the inclusion rate of each feedstuff by its respective value obtained from supplementary Table S1 and summing these values. [4] β-mannanase activity units (U, IU, or TMU) represent degradation rate of mannose-containing substrate per minute under specific reaction conditions (e.g., pH, temperature, and substrate sources) [22,24]. [5] Direct comparison of β-mannanase inclusion levels across studies was not possible, as β-mannanase activity units (U/kg, IU/kg, or TMU/kg) were determined under non-standardized reaction conditions [25,26], and some studies reported inclusion solely as a % without enzyme activity. Thus, direct comparison based on β-mannanase units across studies was not considered in this review. [6] The chemical composition was an analyzed value.

Supplementary Table S4. Growth performance in pigs fed high β-mannan diets with β-mannanase[1, 2].

IBW (kg)	NSP composition[3] (% in DM diet)		Experimental period (d)	ME (kcal/kg)	Number of replicates	β-mannanase (% or unit/kg)[4, 5]	Growth performance (% change)			Reference
	β-mannans	Total NSP (tNSP)					ADG	ADFI	G:F	
5.8	0.50	10.9	42	3409	8	0.025%	0.79	−0.18	1.01	[48]
5.9	0.69	10.8	42	3440	6	400 U/kg	11.83	3.05	8.58	[49]
6.0	1.97	11.6	42	N/A	10	0.01%	5.67 **	−0.32	5.90 **	[19]
						0.02%	9.36 **	0.63	8.54 **	
						0.03%	9.11 **	1.27	7.76 **	
6.2	0.53	11.3	37	3383	8	800 TMU/kg	1.39	−9.33	12.90 **	[20]
6.2	0.52	11.7	42	3337	6	800 TMU/kg	8.41	6.1	1.41	[50]
6.2	0.54	10.0	42	3400	10	800 U/kg	0.00	0.28	0.00	[51]
						10,000 U/kg	3.42	−0.71	4.48 **	
6.9	0.59	11.4	35	3375	8	96,000 U/kg	5.08	−3.76	−1.73	[52]
8.7[6]	0.53	12.4	42	3360	8	800 U/kg	7.46	2.58	6.12	[53]
9.0	0.87	16.1	27	3322	6	70,000 U/kg	−2.34 **	−5.70	3.56	[54]
15.7	0.72	14.3	11	3340	12	200 U/kg	−0.87	1.63	−2.13	[22]
						400 U/kg	−1.74	−2.72	2.13	
						600 U/kg	−2.61	−0.54	−2.13	
15.4	0.66	13.6	10	3390	24	400 U/kg	−5.16	−4.91	0.00	
						600 U/kg	1.76	−2.58	4.69	
17.6	0.74	14.7	35	3300	14	400 U/kg	0.51	−2.24	2.97**	[23]
6.3	0.39	8.6	42	N/A	6	0.05%	1.04	−2.90	4.53**	[55]
	0.54	10.9				0.05%	3.71	0.16	3.46**	
13.6	0.62	13.3	21	N/A	10	0.05%	2.76	−1.78	4.75 **	
22.5	0.48	11.6	N/A	N/A	5	0.05%	3.56 **	−0.80	4.15 **	
23.6	0.52	13.0	28	3400 (DE)	6	200 U/kg	14.14 **	−2.28	17.50 **	[56]
						400 U/kg	19.25 **	−2.82	22.50 **	
						600 U/kg	12.63 **	−5.05	17.50 **	
23.5	0.53	14.5	28	3250 (DE)	6	400 U/kg	17.98 **	−2.07	20.00 **	
	0.52	13.0		3400 (DE)			18.75 **	−5.78	25.94 **	
29.6	0.57[7]	13.0	92	3398	7	80,000 U/kg	0.47	0.71	−0.61	[57]
36.2	2.40[7]	13.7	42	3274	4	400 U/kg	3.35 **	−0.14	3.49 **	[58]
	2.66[7]	13.8		3346		400 U/kg	1.98	−0.29	2.27 **	
34.9	2.52[7]	13.8	42	3303	4	400 U/kg	4.47 **	−0.89	5.14 **	[59]
						800 U/kg	6.04 **	−1.33	7.69 **	
45.6	0.61[7]	12.2	42	3303	4	400 U/kg	5.18 **	1.57	3.56 *	
						800 U/kg	7.77 **	3.55	4.08 *	
49.9	3.16	15.6	42	3300	5	400 U/kg	2.46 **	0.15	2.30 **	[60]
				3260		400 U/kg	4.10 **	0.39	3.69 **	
56.0	0.60	14.7	30	3258	10	0.004%	8.24	7.76	0.45	[61]
57.6	0.42	11.2	28	3300	4	200 U/kg	0.71	−0.45	0.94	[62]
						400 U/kg	2.36 **	−0.94	3.14	
						600 U/kg	1.65 **	0.11	1.57	
92.7	0.34	10.0	28	3300	4	200 U/kg	0.39	−0.67	1.05	
						400 U/kg	4.68 **	−0.56	5.23	

						600 U/kg	2.60 **	0.22	2.09
60.5	0.50	12.1	23	3300	4	400 U/kg	1.37	0.88	0.60
	0.56	13.4				400 U/kg	3.14 **	0.67	2.74
86.5	0.46	11.1	29	3300	4	400 U/kg	4.13 **	−3.66	8.46
	0.55	13.4				400 U/kg	2.49 **	0.72	1.53

IBW = initial body weight; DM = dry matter; ME = metabolizable energy; ADG = average daily gain; ADFI = average daily feed intake; G:F = gain to feed ratio; DE = digestible energy. [1] Asterisk marks (*, **) represent statistical tendency ($p < 0.10$) and significant difference ($p < 0.05$), respectively. [2] The percentage change of the ADG, ADFI, and G:F was determined as: [(mean value in the β-mannanase group – mean value in the control group without β-mannanase)/mean value in the control group without β-mannanase] × 100, and all percentage changes were recalculated in this study based on the reported values from the original studies. [3] The dietary contents of both β-mannans and tNSP were determined by multiplying the inclusion rate of each feedstuff by its respective value obtained from supplementary Table S1 and summing these values. [4] β-mannanase activity units (U or TMU) represent degradation rate of mannose-containing substrate per minute under specific reaction conditions (e.g., pH, temperature, and substrate sources) [22,24]. [5] Direct comparison of β-mannanase inclusion levels across studies was not possible, as β-mannanase activity units (U/kg or TMU/kg) were determined under non-standardized assay conditions (e.g., pH, temperature, and substrate sources) [25,26], and some studies reported inclusion solely as a % without enzyme activity. Thus, direct comparison based on β-mannanase units across studies was not considered in this review. [6] Pigs were fed a mycotoxin-contaminated diet containing 150 μg aflatoxin B1 and 1,100 μg deoxynivalenol per kg diet. [7] The chemical composition was an analyzed value.

Supplementary Table S5. Study-level influence diagnostics for the meta-analysis of digesta viscosity in the small intestine of chickens.

Slope (β-mannans) when excluded each study	Standard error	*p*-value	Cook's distance[1]	Restricted likelihood distance[2]	Number excluded	Number remaining	Reference[3]
-0.585	0.208	<0.05				54	
-0.372	0.188	0.056	0.458	5.287	12	42	[11]
-0.621	0.220	<0.05	0.014	0.142	3	51	[13]
-1.138	0.291	<0.05	3.011	4.745	8	46	[14]
-0.614	0.219	<0.05	0.008	0.137	3	51	[15]
-0.584	0.242	<0.05	0.002	0.177	18	36	[16]
-0.606	0.220	<0.05	0.005	0.095	2	52	[17]
-0.121	0.109	0.274	2.273	92.626	6	48	[10]
-0.866	0.237	<0.05	1.219	6.274	2	52	[18]

[1] Cook's distance are case-deletion influence diagnostics; higher values indicate greater influence of that study on the slope of dietary β-mannans. [2] Restricted likelihood distance is case-deletion influence diagnostics; higher values indicate greater influence of that study on the overall model. [3] Reference number of the study excluded from the meta-analysis of digesta viscosity in the small intestine of chicken in that row.

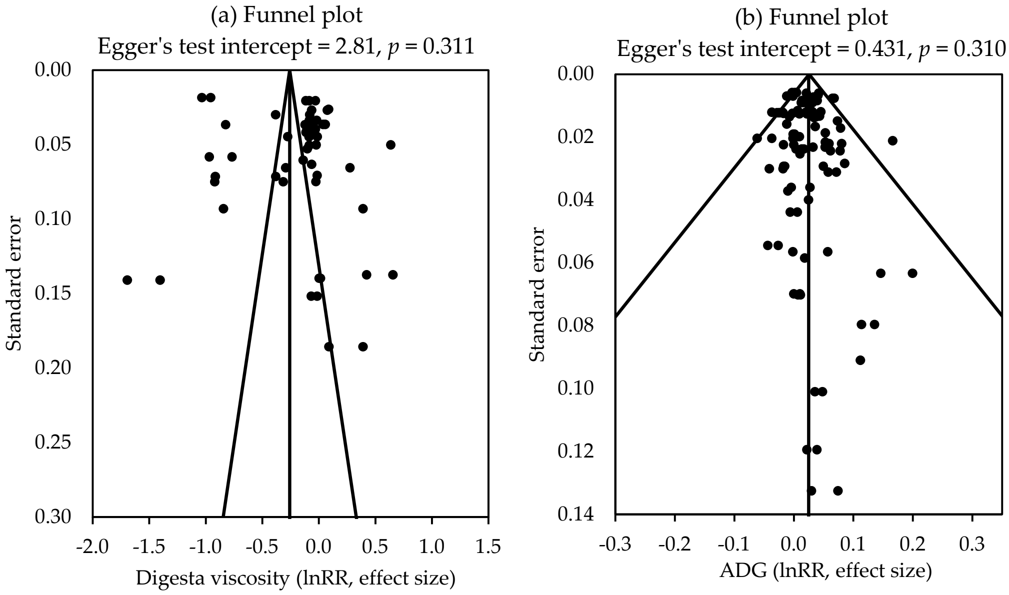

Supplementary Figure S1. Funnel plots for assessing publication bias in the meta-analyses of (a) digesta viscosity in the small intestine and (b) average daily gain (ADG) of chickens, both expressed as log response ratio (lnRR) plotted against its standard error.

References

1. Knudsen, K.E.B. Carbohydrate and lignin contents of plant materials used in animal feeding. *Anim. Feed Sci. Technol.* **1997**, *67*, 319-338.
2. Jaworski, N.W.; Lærke, H.N.; Bach Knudsen, K.E.; Stein, H.H. Carbohydrate composition and in vitro digestibility of dry matter and nonstarch polysaccharides in corn, sorghum, and wheat and coproducts from these grains. *J. Anim. Sci.* **2015**, *93*, 1103-1113.
3. Choct, M. Feed non-starch polysaccharides: chemical structures and nutritional significance. *Feed Milling Int.* **1997**, *191*, 13-26.
4. Gutierrez, N.A.; Serão, N.V.L.; Kerr, B.J.; Zijlstra, R.T.; Patience, J.F. Relationships among dietary fiber components and the digestibility of energy, dietary fiber, and amino acids and energy content of nine corn coproducts fed to growing pigs. *J. Anim. Sci.* **2014**, *92*, 4505-4517.
5. Rodehutscord, M.; Rückert, C.; Maurer, H.P.; Schenkel, H.; Schipprack, W.; Bach Knudsen, K.E.; Schollenberger, M.; Laux, M.; Eklund, M.; Siegert, W. Variation in chemical composition and physical characteristics of cereal grains from different genotypes. *Arch. Anim. Nutr.* **2016**, *70*, 87-107.
6. Knudsen, K.E.B. Fiber and nonstarch polysaccharide content and variation in common crops used in broiler diets. *Poult. Sci.* **2014**, *93*, 2380-2393.
7. Slominski, B.A.; Campbell, L.D.; Guenter, W. Carbohydrates and dietary fiber components of yellow-and brown-seeded canola. *Journal of Agricultural and Food Chemistry* **1994**, *42*, 704-707.
8. Saittagaroon, S.; Kawakishi, S.; Namiki, M. Characterisation of polysaccharides of copra meal. *Journal of the Science of Food and Agriculture* **1983**, *34*, 855-860.
9. Kiarie, E.G.; Steelman, S.; Martinez, M.; Livingston, K. Significance of single β-mannanase supplementation on performance and energy utilization in broiler chickens, laying hens, turkeys, sows, and nursery-finish pigs: a meta-analysis and systematic review. *Transl. Anim. Sci.* **2021**, *5*, txab160.
10. Caldas, J.V.; Vignale, K.; Boonsinchai, N.; Wang, J.; Putsakum, M.; England, J.A.; Coon, C.N. The effect of β-mannanase on nutrient utilization and blood parameters in chicks fed diets containing soybean meal and guar gum. *Poult. Sci.* **2018**, *97*, 2807-2817.
11. Lee, J.T.; Bailey, C.A.; Cartwright, A.L. β-mannanase ameliorates viscosity-associated depression of growth in broiler chickens fed guar germ and hull fractions. *Poult. Sci.* **2003**, *82*, 1925-1931.
12. Sundu, B.; Kumar, A.; Dingle, J. Response of broiler chicks fed increasing levels of copra meal and enzymes. *Int. J. Poult. Sci.* **2006**, *5*, 13-18.
13. Mehri, M.; Adibmoradi, M.; Samie, A.; Shivazad, M. Effects of β-mannanase on broiler performance, gut morphology and immune system. *Afr. J. Biotechnol.* **2010**, *9*, 6221.
14. Gharaei, M.A.; Dastar, B.; Nameghi, A.H.; Tabar, G.H.; Shargh, M.S. Effects of guar meal with and without β-mannanas enzyme on performance and immune response of broiler chicks. *Int. Res. J. Appl. Basic Sci.* **2012**, *3*, 2785-2793.
15. Hosseindoust, A.; Lee, S.; Nho, W.G.; Song, Y.H.; Shin, J.S.; Ingale, S.L.; Rathi, P.C.; Choi, J.; Chae, B.; Kim, J. A dose-response study to evaluate the effects of pH-stable β-mannanase derived from *Trichoderma citrinoviride* on growth performance, nutrient retention, and intestine morphology in broiler chickens. *Ital. J. Anim. Sci.* **2019**, *18*, 147-154.
16. Latham, R.E.; Williams, M.P.; Walters, H.G.; Carter, B.; Lee, J.T. Efficacy of β-mannanase on broiler growth performance and energy utilization in the presence of increasing dietary galactomannan. *Poult. Sci.* **2018**, *97*, 549-556.
17. Barekatain, R.; Hall, L.; Chrystal, P.V.; Fickler, A. Nutrient utilisation and growth performance of broiler chickens fed standard or moderately reduced dietary protein diets with and without β-mannanase supplementation. *Anim. Nutr.* **2024**, *19*, 131-138.
18. Lee, E.C.; Kim, K.H.; Mugeniwayesu, C.; Jeong, Y.J.; Kil, D.Y. Effects of dietary β-mannanase supplementation in low-energy and low-protein diets containing high-mannan ingredients on growth performance, meat quality, intestinal morphology, and liver health in broiler chickens. *Anim. Biosci.* **2025**, *39*, 250545.
19. Tajudeen, H.; Mun, J.Y.; Ha, S.; Hosseindoust, A.; Kinara, E.; Lokhande, A.; Ingale, S.L.; Kim, J.S. The immunomodulatory activities of a thermostable galacto-mannanase and their impact on the performance of weaned piglets. *Anim. Feed Sci. Technol.* **2025**, *319*, 116186.
20. Baker, J.T.; Deng, Z.; Sokale, A.; Frederick, B.; Kim, S.W. Nutritional and functional roles of β-mannanase on intestinal health and growth of newly weaned pigs fed two different types of feeds. *J. Anim. Sci.* **2024**, *102*, skae206.
21. Tiwari, U.P.; Chen, H.; Kim, S.W.; Jha, R. Supplemental effect of xylanase and mannanase on nutrient digestibility and gut health of nursery pigs studied using both in vivo and in vitro models. *Anim. Feed Sci. Technol.* **2018**, *245*, 77-90.
22. Jang, K.B.; Kim, Y.I.; Duarte, M.E.; Kim, S.W. Effects of β-mannanase supplementation on intestinal health and growth of nursery pigs. *J. Anim. Sci.* **2024**, *102*, skae052.
23. Chen, H.; Garavito-Duarte, Y.; Kim, Y.I.; Zhang, S.; Kim, S.W. Effects of β-mannanase on intestinal health and growth performance of nursery to growing pigs fed diets with DDGS. *J. Anim. Sci.* **2025**, *103*, skaf238.

24. Williams, M.P.; Brown, B.; Rao, S.; Lee, J.T. Evaluation of beta-mannanase and nonstarch polysaccharide-degrading enzyme inclusion separately or intermittently in reduced energy diets fed to male broilers on performance parameters and carcass yield. *J. Appl. Poult. Re* **2014**, *23*, 715-723.
25. Adeola, O.; Cowieson, A.J. Board-invited review: opportunities and challenges in using exogenous enzymes to improve nonruminant animal production. *J. Anim. Sci.* **2011**, *89*, 3189-3218.
26. Choct, M. Enzymes for the feed industry: past, present and future. *World's Poult. Sci. J.* **2006**, *62*, 5-16.
27. Zhang, X.; Wang, B.; Ban, Z.; Wang, M.; Wang, Y.; Wang, X.; Guo, Y. Impact of mannanase on broiler performance, intestinal health, and energy utilization with varying soybean meal levels. *Anim. Adv.* **2025**, *2*, e012.
28. Zou, J.; Zheng, P.; Zhang, K.; Ding, X.; Bai, S. Effects of exogenous enzymes and dietary energy on performance and digestive physiology of broilers. *J. Anim. Sci. Biotechnol.* **2013**, *4*, 14.
29. Li, Y.; Chen, X.; Chen, Y.; Li, Z.; Cao, Y. Effects of β-mannanase expressed by Pichia pastoris in corn-soybean meal diets on broiler performance, nutrient digestibility, energy utilization and immunoglobulin levels. *Anim. Feed Sci. Technol.* **2010**, *159*, 59-67.
30. Kong, C.; Lee, J.H.; Adeola, O. Supplementation of β-mannanase to starter and grower diets for broilers. *Can. J. Anim. Sci.* **2011**, *91*, 389-397.
31. Azarfar, A. Effect of hemicell enzyme on the performance, growth parameter, some blood factors and ileal digestibility of broiler chickens fed corn/soybean-based diets. *J. Cell Anim. Biol.* **2013**, *7*, 85-91.
32. Ferreira Jr, H.C.; Hannas, M.I.; Albino, L.F.T.; Rostagno, H.S.; Neme, R.; Faria, B.D.; Xavier Jr, M.L.; Rennó, L.N. Effect of the addition of β-mannanase on the performance, metabolizable energy, amino acid digestibility coefficients, and immune functions of broilers fed different nutritional levels. *Poult. Sci.* **2016**, *95*, 1848-1857.
33. Jackson, M.E.; Geronian, K.; Knox, A.; McNab, J.; McCartney, E. A dose-response study with the feed enzyme beta-mannanase in broilers provided with corn-soybean meal based diets in the absence of antibiotic growth promoters. *Poult. Sci.* **2004**, *83*, 1992-1996.
34. Mussini, F.J.; Coto, C.A.; Goodgame, S.D.; Lu, C.; Karimi, A.J.; Lee, J.H.; Waldroup, P.W. Effect of ß-mannanase on broiler performance and dry matter output using corn-soybean meal based diets. *Int. J. Poult. Sci.* **2011**, *10*, 778-781.
35. Zou, X.T.; Qiao, X.J.; Xu, Z.R. Effect of β-mannanase (Hemicell) on growth performance and immunity of broilers. *Poult. Sci.* **2006**, *85*, 2176-2179.
36. Latham, R.E.; Williams, M.; Smith, K.; Stringfellow, K.; Clemente, S.; Brister, R.; Lee, J.T. Effect of β-mannanase inclusion on growth performance, ileal digestible energy, and intestinal viscosity of male broilers fed a reduced-energy diet. *J. Appl. Poult. Re* **2016**, *25*, 40-47.
37. Marçal, B.V.; Silva, R.L.; Maria, L.P.; dos Santos Araújo, R.; Dourado, L.R.B.; da Silva, M.K.; Sakomura, N.K. Effects of beta-mannanase supplementation on growth performance, digestibility, intestinal morphometry, and cecal microbiota in broiler chickens. *Livest. Sci.* **2025**, 105817.
38. Yaqoob, M.U.; Yousaf, M.; Khan, M.I.; Wang, M. Effect of β-mannanase supplementation on growth performance, ileal digestibility, carcass traits, intestinal morphology, and meat quality in broilers fed low-ME diets. *Animals* **2022**, *12*, 1126.
39. Zhang, L.; Huan, H.; Zhang, K.; Tu, Y.; Yan, J.; Zhang, H.; Xi, Y. The effects of β-mannanase supplementation on growth performance, digestive enzyme activity, cecal microbial communities, and short-chain fatty acid production in broiler chickens fed diets with different metabolizable energy levels. *J. Anim. Sci.* **2024**, *102*, skae184.
40. Ahsan, T.; Tahir, M.; Khan, R.U.; Ahmad, S.; Khan, N.A.; Konca, Y.; Alhidary, I.A.; Abdelrahman, S.; Tarricone, S. Impact of varying levels of soy hulls and β-mannanase enzyme supplementation on growth performance, carcass characteristics, nutrient utilization and blood biochemical profile in broiler chickens. *Ital. J. Anim. Sci.* **2024**, *23*, 1551-1560.
41. Chegeni, A.; Torki, M.; Kamyab, A. Effects of β-mannanase-based enzyme in corn-soy and corn-soy-canola diets on broiler performance. *J. Appl. Anim. Res.* **2011**, *39*, 261-268.
42. Hashim, M.M.; El-Safty, S.A.; El-Eraqi, K.G.; El-Sherif, H.M.R.; Azza, T.; Marwa, K.; Ibrahim, H.M.; Abdelhady, A.Y.M. Effect of β-mannanase enzymes supplementation to energy deficient diets on productive performance, physiological and carcass traits of broilers. *Int. J. Poult. Sci.* **2020**, *19*, 455-466.
43. Kim, E.; Choct, M.; Fickler, A.; Pasquali, G.A.M.; Hall, L.; Crowley, T.M.; Sharma, N.K. Supplementation of β-mannanase alone or in combination with xylanase and β-glucanase enhanced growth performance, non-starch polysaccharide degradation, and gastrointestinal environment of broilers offered wheat-based diets. *Anim. Nutr.* **2025**, *23*, 429-437.
44. Chuppava, B.; Abd El-Wahab, A.; Schiel, B.; Ratert, C.; Reckels, B.; Visscher, C.; Kamphues, J.; Lingens, J.B. Impacts of mannanase supplementation in guar meal by-product on broiler chickens performance, foot pad health and selected organ traits: a pilot study. *Eur. Poult. Sci.* **2022**, *86*, 1-18.
45. de Souza, M.; Eeckhaut, V.; Goossens, E.; Ducatelle, R.; Van Nieuwerburgh, F.; Poulsen, K.; Baptista, A.A.S.; Bracarense, A.P.F.R.L.; Van Immerseel, F. Guar gum as galactomannan source induces dysbiosis and reduces performance in broiler chickens and dietary β-mannanase restores the gut homeostasis. *Poult. Sci.* **2023**, *102*, 102810.

46. El-Masry, K.N.; Ragaa, N.M.; Tony, M.A.; El-Banna, R.A. Effect of dietary inclusion of guar meal with or without β-mannanase supplementation on broiler performance and immunity. *Pak. J. Nutr.* **2017**, *16*, 341-350.
47. Daskiran, M.; Teeter, R.G.; Fodge, D.; Hsiao, H.Y. An evaluation of endo-β-D-mannanase (Hemicell) effects on broiler performance and energy use in diets varying in β-mannan content. *Poult. Sci.* **2004**, *83*, 662-668.
48. Carr, S.N.; Allee, G.L.; Rincker, P.J.; Fry, R.S.; Boler, D.D. Effects of endo-1, 4-β-d-mannanase enzyme (Hemicell HT 1.5×) on the growth performance of nursery pigs. *Prof. Anim. Sci.* **2014**, *30*, 393-399.
49. Jang, K.B.; Kim, Y.; Ahn, J.; Lee, J.I.; Park, S.; Choe, J.; Kim, Y.; Lee, J.H.; Kyoung, H.; Song, M. Dietary β-mannanase reduced post-weaning diarrhea of pigs by positively modulating gut microbiota and attenuating systemic immune responses. *Anim. Microbiom.* **2024**, *6*, 59.
50. Choi, H.; Deng, Z.; Sokale, A.; Von Heimendahl, E.; Amerah, A.M.; Kim, S.W. Investigation of nutritional and functional roles of β-mannanase on intestinal health and growth performance of nursery pigs fed low-cost formulated diets with high phytase inclusion. *J. Anim. Sci.* **2026**, *104*, skag038.
51. Acosta, J.P.; Lee, S.A.; Fickler, A.; Stein, H.H. Tolerance of weanling pigs and effects on growth performance of supplementing corn-soybean meal-based diets with graded levels of a novel exogenous β-mannanase. *Transl. Anim. Sci.* **2025**, *9*, txaf061.
52. Jang, J.-C.; Kim, K.H.; Jang, Y.D.; Kim, Y.Y. Effects of dietary β-mannanase supplementation on growth performance, apparent total tract digestibility, intestinal integrity, and immune responses in weaning pigs. *Animals* **2020**, *10*, 703.
53. Garavito-Duarte, Y.R.; Son, J.; Weaver, A.C.; Kim, S.W. Functional efficacies of humate and β-mannanase against Aflatoxin B1 and Deoxynivalenol in the diets for nursery pigs. *Toxins* **2025**, *17*, 456.
54. Jones, C.K.; Bergstrom, J.R.; Tokach, M.D.; DeRouchey, J.M.; Goodband, R.D.; Nelssen, J.L.; Dritz, S.S. Efficacy of commercial enzymes in diets containing various concentrations and sources of dried distillers grains with solubles for nursery pigs. *J. Anim. Sci.* **2010**, *88*, 2084-2091.
55. Pettey, L.A.; Carter, S.D.; Senne, B.W.; Shriver, J.A. Effects of beta-mannanase addition to corn-soybean meal diets on growth performance, carcass traits, and nutrient digestibility of weanling and growing-finishing pigs. *J. Anim. Sci.* **2002**, *80*, 1012-1019.
56. Lv, J.N.; Chen, Y.Q.; Guo, X.J.; Piao, X.S.; Cao, Y.H.; Dong, B. Effects of supplementation of β-mannanase in corn-soybean meal diets on performance and nutrient digestibility in growing pigs. *Asian-Australas J. Anim. Sci.* **2013**, *26*, 579.
57. Jacela, J.Y.; Dritz, S.S.; DeRouchey, J.M.; Tokach, M.D.; Goodband, R.D.; Nelssen, J.L. Effects of supplemental enzymes in diets containing distillers dried grains with solubles on finishing pig growth performance. *Prof. Anim. Sci.* **2010**, *26*, 412-424.
58. Kim, J.S.; Ingale, S.L.; Lee, S.H.; Kim, K.H.; Lee, J.H.; Chae, B.J. Effects of energy levels of diet and β-mannanase supplementation on growth performance, apparent total tract digestibility and blood metabolites in growing pigs. *Anim. Feed Sci. Technol.* **2013**, *186*, 64-70.
59. Kim, J.S.; Ingale, S.L.; Hosseindoust, A.R.; Lee, S.H.; Lee, J.H.; Chae, B.J. Effects of mannan level and β-mannanase supplementation on growth performance, apparent total tract digestibility and blood metabolites of growing pigs. *Animal* **2017**, *11*, 202-208.
60. Jeon, S.M.; Hosseindoust, A.; Choi, Y.H.; Kim, M.J.; Kim, K.Y.; Lee, J.H.; Kil, D.Y.; Kim, B.G.; Chae, B.J. Comparative standardized ileal amino acid digestibility and metabolizable energy contents of main feed ingredients for growing pigs when adding dietary β-mannanase. *Anim. Nutr.* **2019**, *5*, 359-365.
61. Liu, F.; Li, J.; Ni, H.; Azad, M.A.K.; Mo, K.; Yin, Y. The effects of phytase and non-starch polysaccharide-hydrolyzing enzymes on trace element deposition, intestinal morphology, and cecal microbiota of growing–finishing pigs. *Animals* **2023**, *13*, 549.
62. Yoon, S.Y.; Yang, Y.X.; Shinde, P.L.; Choi, J.Y.; Kim, J.S.; Kim, Y.W.; Yun, K.; Jo, J.K.; Lee, J.H.; Ohh, S.J. Effects of mannanase and distillers dried grain with solubles on growth performance, nutrient digestibility, and carcass characteristics of grower-finisher pigs. *J. Anim. Sci.* **2010**, *88*, 181-191.
